# Oral iron-induced stunting is associated with increased *Christensenella* abundance in a piglet model for human infants

**DOI:** 10.1007/s00394-026-04082-9

**Published:** 2026-08-27

**Authors:** Munawar Abbas, Maria Batool, Zeynep Hayirli, Carlos Poveda, Kimon-Andreas Karatzas, Gemma E. Walton, Umer Zeeshan Ijaz, Simon C. Andrews, Marie C. Lewis

**Affiliations:** 1 Food and Nutritional Sciences, University of Reading, Whiteknights, Reading UK; 2 Teagasc Food Research Centre, Moorepark, Fermoy, Co. Cork Ireland; 3 Mazumdar-Shaw Advanced Research Centre, University of Glasgow, Glassgow, UK; 4 Department of Molecular and Clinical Cancer Medicine, University of Liverpool, Liverpool, UK; 5 College of Science and Engineering, University of Galway, Galway, Ireland; 6 School of Biological Sciences, University of Reading, Whiteknights, Reading UK

**Keywords:** Piglet models, Iron supplementation, Gut microbiota, Iron deficiency anaemia, Iron deficiency, Immune development

## Abstract

**Purpose:**

Iron deficiency anaemia (IDA) affects around 40% of < 5-year-olds globally. Unfortunately, IDA treatment by oral iron intervention often causes diarrhoea, thought to be linked to iron-induced changes in the gut microbiota. Piglets are valuable preclinical models for human infants since they share many physiological characteristics, have similar gut microbial populations and, if left untreated with iron, reliably develop IDA. We hypothesised that IDA and different forms of iron supplementation (oral and injection) would have differential effects on the gut microbiota that would consequently differentially impact immune and metabolic development during infancy.

**Methods:**

24 one-day-old piglets were litter-matched into 4 treatment groups (n = 6): control (no iron); intramuscular iron (IM, 200mg Fe); oral iron (200mg FeSO_4_/kg feed); IM+oral iron combined.

**Results:**

As expected, the iron-free treatment piglets developed IDA. This was combined with stunted growth and corresponding changes in plasma metabolites and immune parameters. However, although all 3 iron treatments prevented the development of IDA, piglets receiving oral iron were also stunted (compared to those which received IM iron; 14–19% weight-gain reduction over 27 days,* p*<0.05). This stunting was linked to significant changes in the expression of immune-associated proteins in the gut and specific bacterial genera, but not plasma metabolites. Importantly, the correlation between oral-iron treatment, reduced weight gain and increased *Christensenella* abundance provides novel evidence linking iron-induced stunting with *Christensenella*.

**Conclusion:**

The findings relayed here thus raise health concerns for infants receiving iron-fortified formula milk and/or those in LMICs enrolled in blanket oral iron supplementation programs.

**Supplementary Information:**

The online version contains supplementary material available at https://doi.org/10.1007/s00394-026-04082-9.

## Background

Iron deficiency anaemia (IDA) affects ~1.2 billion individuals globally and is the most prevalent form of malnutrition in young children [1, 2]. IDA is thus a major public health concern, particularly in low- and middle-income countries (LMICs) [3, 4]. Infant IDA occurs when blood haemoglobin (Hb) levels are reduced to  < 105 or < 100 g/L in 4–6 and 9-month old human infants, respectively, and arises due to a reduced rate of erythropoiesis resulting from iron deficiency [5–7]. The primary cause of infant IDA is high iron demand during the rapid growth and development of infants [8, 9], combined with an iron-deficient diet and low iron endowments from the mother during pregnancy [10, 11]. Indeed, during IDA the use of iron for essential functions (e.g., oxygen transport) is prioritized over other processes such as those necessary for growth and development [12]. This can lead to reductions in muscle mass [13] and bone density [14] which contribute to stunted growth. Stunted children are at increased risk of poor physical and cognitive development, as well as poor health outcomes in later life [15]. It is estimated that the annual costs of IDA in infants and young children in India alone accounts for 8.3 million disability-adjusted life years (DALYs) representing a production loss of approximately US$ 2.4 billion (1.3% of GDP) [16].

Infants require at least 0.5 – 11 mg of iron per day, of which 75% is used for growth. However, breast milk contains only ~ 0.3 mg of iron per litre and just 50% of this is absorbed [17]. Therefore, once the infant’s maternal iron endowments have been depleted (at about 6 months of age), substantial amounts of exogenous iron are required [18]. The most commonly applied treatment for IDA is oral iron supplementation (e.g., ferrous sulphate, ferrous gluconate and ferrous fumarate) [19], which often causes diarrhoea, nausea and increased risk of enteric infection [20]. Moreover, universal iron supplementation policies in areas with a high prevenance (~ 40–50%) of IDA [21] result in the unnecessary delivery of excess oral iron to significant numbers of iron-replete infants. In more affluent societies, infant formula milk is often fortified with iron (~ 10–14 mg/L) [22, 23], which can similarly induce severe diarrhoea [24, 25]. Only around 10% of oral iron supplements are absorbed and thus most remain in the digestive tract where they can negatively impact the composition and metabolism of the gut microbiota, and increase pathogenic potential of enteric pathogens [26], which could contribute to the observed increased risks of enteric infection and diarrhoea [27]. Parenteral iron formulae (e.g., iron dextran, iron sucrose and ferric carboxymaltose) are used as rapid-action alternatives to oral iron supplementation; these are administered through an intramuscular (IM) injection or intravenous (IV) drip [28]. Although such treatments bypass the gut, they are more likely to result in iron overload, nausea and dizziness [29], and can cause toxicity effects in some organs, including the heart [30].

Although only a small proportion of colonic luminal iron is bioavailable to gut microbes [31], this limited iron environment triggers the bacteria to synthesise siderophores, small, high-affinity iron-chelating compounds, which significantly enhance iron uptake by components of the gut microbiota [32]. However, we are not aware of any strong evidence that oral iron supplements raise availability of iron to the gut microbiota (i.e. direct evidence of suppression of iron restriction by the host). Iron is a key requirement for nearly all gut microbes but increased iron has been associated with significant increases in the abundance of *Enterobacteriacea,* including enteropathogenic *Escherichia coli* (EPEC) [33], and increased pathogenic potential of *Salmonella* Typhimurium, for example, by triggering the expression of virulence genes including those for toxin production, chemoattraction to epithelial surfaces and adhesion mechanisms [26]. This is in conjunction with significant decreases in numbers of beneficial bacteria, including lactobacilli [34]. Changes in the metabolic activity of gut microbiota populations in response to iron availability have also been reported, evidenced by alterations in the production of short chain fatty acids (SCFAs), major gut-bacterial metabolites that play crucial roles in both gut and overall health [35]. However, the effects of oral iron on SCFA production remain unclear; both IDA and excess iron availability have been linked with reductions in SCFA concentrations [7, 36] while increases have also been reported [37, 38]. These findings suggest that excessive iron supplementation could have negative impacts on both the composition and metabolic activity of the gut microbiota, potentially promoting the growth of gut pathogens and reducing the abundance of beneficial bacterial populations. Although the effects of iron supplementation on the infant gut microbiota are less clear, recent meta-analysis of four independent studies demonstrated that *Bifidobacterium* populations are consistently less abundant following oral iron supplementation [39]. This highlights the necessity for careful consideration of the risks and benefits of iron supplementation programs, particularly for vulnerable populations in low-hygiene environments, and the importance of monitoring the effects of different forms of iron supplementation on the gut microbiota.

Pigs are valuable, preclinical models for humans [40] since they share many features of gastrointestinal physiology, immunology, microbiology, diet and pathology [41–45], and are also more outbred than rodent models which better reflects human heterogeneity. Piglets are particularly useful for exploring the impact of early-life nutrition on physiological development since their precocial self-sufficiency enables early separation from their mothers, permitting tight control of pre-weaned infant feed intake. Additionally, newborn piglets suffer from low-iron endowments due to large litter sizes. This effect is exacerbated by subsequent rapid growth such that piglets reliably develop IDA within a week or two of birth, unless iron treatment is applied [46, 47] (see our review [23]). Additionally, there are similarities in iron-dependent blood parameters in pigs and humans in response to IDA [48]. We have also previously demonstrated that the metabolic profiles in blood, urine and various pig organs are qualitatively comparable to those in humans [49]. Furthermore, the gut microbiota in humans and pigs are considerably more stable over time than in rodent models [50], and pigs and humans share similar gut microbiota diversities, heterogeneity and dominant phyla, including Firmicutes and Bacteroides [51]. Piglets have also been shown to exhibit responses to dietary interventions that are more similar to humans than those of rodents [52]. Overall, the piglet model provides a unique and valuable opportunity to explore how the infant gut microbiota, host-microbe interactions and the gut immune system respond to IDA and to different types of iron treatment, under conditions more closely resembling human infant physiology than those provided by rodent models.

The aim of this study was to explore the effects of IDA and different types of iron treatment on physiological development at the pre-weaned infant stage using piglets as models for human infants. Since oral-iron supplementation increases iron availability to the gut microbiota, we hypothesised that different forms of iron treatment (oral delivery and IM injection) would have differential effects on the developing gut microbiota and on the subsequent development of the gut mucosal immune system. Any such effects could have major health implications since the development of the immune system influences functionality and infection susceptibility in later life [53, 54].

## Methods

### The piglet model

**Fig. 1:**
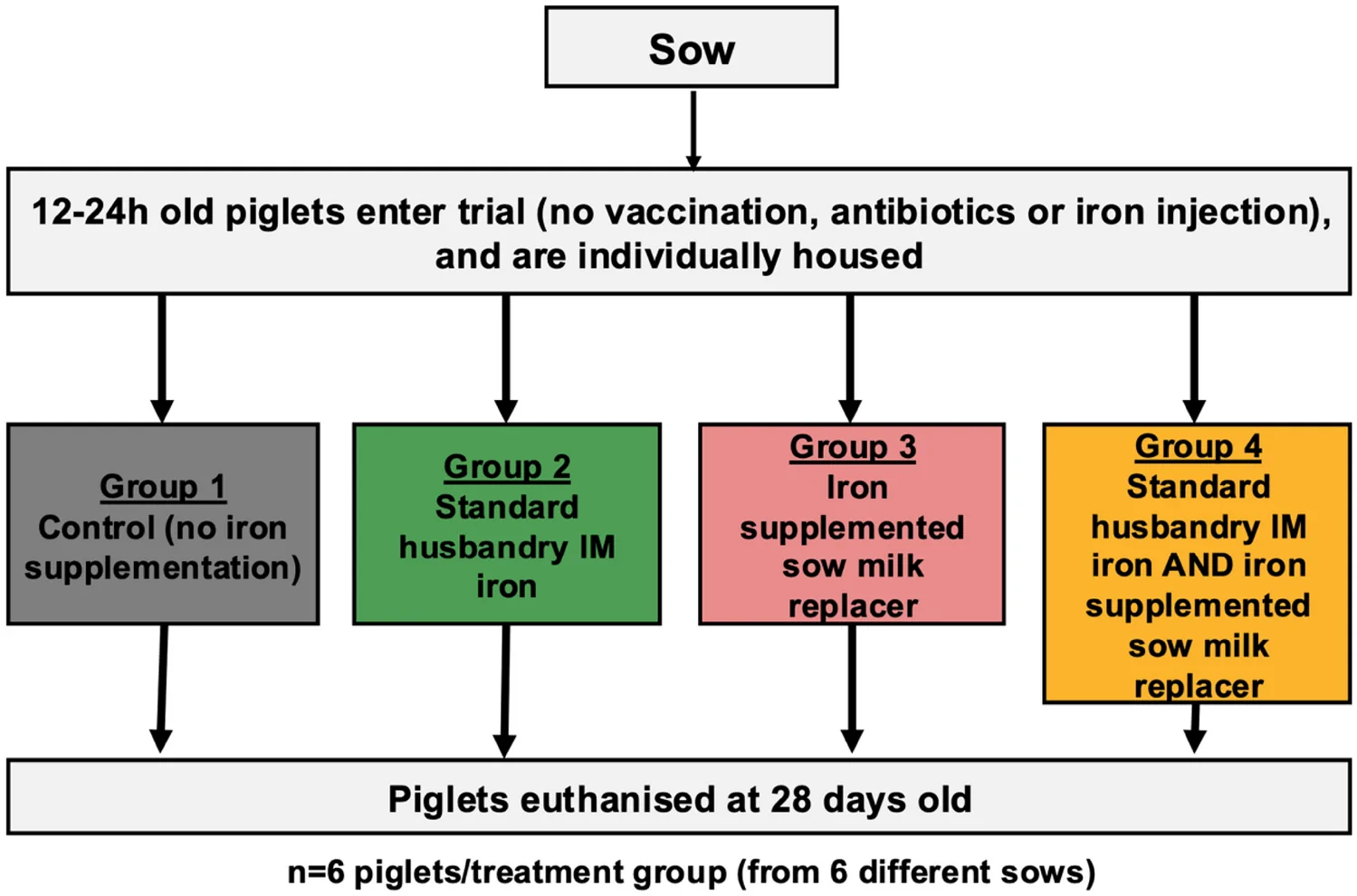
Schematic representation of the iron-treatment regimes applied during the piglet trial. n = 6 litter-matched piglets per treatment group

Animal housing and experimental procedures were performed at the Centre for Dairy Research (CEDAR), University of Reading under UK Home Office licence (PCE914CF), adhered to ARRIVE guidelines and were approved by the Reading Animal Welfare and Ethical Review Body (AWERB). Following birth, piglets were initially left with their mothers for 12–24 h to ensure adequate colostrum intake. At 1 day old, piglets were transported to CEDAR and maintained at 30 °C on wood shavings, provided with heat lamps under a 12 h light/dark cycle and had free access to fresh drinking water. To help reduce the time taken for piglets to learn to drink from bowls, they were initially housed in two groups (receiving either: oral iron at a concentration of 200 mg Fe/kg feed, n = 12; or no oral iron, n=12) to facilitate learning through emulation. Throughout the trial the only source of food was an iron-free bovine-based sow milk replacer (SMR, Table 1) (Withernay Ltd, UK), similar to infant formula milk, which was consumed by all piglets. The amount of SMR consumed according to mean weight of the piglets (measured twice weekly) was as per the manufacturer’s recommendations and is provided in Table 2. Piglets received the same amount of feed (at 20% concentration) irrespective of treatment, and consumed all feed provided to them following the initial day of acclimatisation where feed consumption was sporadic. Commercially, creep feed is provided to piglets from 14 days old to supplement sow milk. However, this contains iron due to the ingredients used (for example fish-meal, soya and grains) and so creep feed was not provided during this trial to ensure tight control of iron consumption. To assess the impact of IDA and different types of iron supplementation, 24 healthy Large White F1 hybrid (Duroc boar) piglets (4 from each of 6 sows) were allocated into four litter- and sex-matched (3 male & 3 female) treatment groups at 2 days old. The treatments applied were: 1, IDA (no iron treatment); 2, intramuscular (IM) injection (200 mg Fe as iron dextran) (control); 3, iron supplemented (FeSO_4_) sow-milk replacer (SMR) (oral iron at 200 mg/kg dry feed); 4, IM (200 mg Fe) and oral iron (200 mg/kg dry feed) combination (Fig. 1). Litter-matching accounted for the genetic variability between groups, enabling studies to be conducted with fewer animals while maintaining statistical power [55, 56]. Prior to their arrival at CEDAR, the piglets did not receive vaccinations, antibiotics or iron treatments. During the trial, piglets were housed in adjacent, mesh-walled pens to permit social interactions and bacterial transfer (to eliminate any ‘pen effects’), whilst preventing non-autologous coprophagy and ensuring tight dietary control. Piglets were initially fed (either iron supplemented or non-supplemented) with 10% SMR (in water) every 4 h (five feeds per day). The SMR concentration was increased to 15% and then to 20% (standard concentration, four feeds per day) over the following two days in order to reduce the risk of diarrhoea [57]. Following twice weekly weight measurements, the amount of feed and FeSO_4_ were increased accordingly to ensure optimal nutrition, with amounts of feed provided based on the manufacturer’s recommendations. Weekly blood samples were collected from the jugular vein into EDTA vacutainers (BD Vacutainer^®^) and haemoglobin (Hb) was quantified weekly to monitor IDA status using a portable Hb meter (HemoCue^®^ 201+, HemoCue AB^©^). At 28 days of age, the piglets were euthanised by injection of an overdose of 20% pentobarbital sodium BP (Dolethal, Vetoquinol Ltd, Northamptonshire, UK). At post-mortem, faecal samples were collected directly from the rectum, and blood and urine were collected from the heart and bladder, respectively, using a syringe equipped with a needle. Additionally, samples of proximal and distal jejunum, along with descending colon, were collected, mounted in optimal cutting temperature (OCT) embedding compound (Thermo Scientific, UK) and snap-frozen in 2-methylbutane (Fisher Scientific Ltd, UK) over liquid nitrogen. All the above samples were then stored at − 80 °C. No piglets were excluded from the trial.

**Table 1:** Sow milk replacer (SMR) composition

Ingredient	Composition (%)
Fat filled whey (Palm and Coconut oil)	40.0
Skimmed milk	22.0
When protein concentrate (35%)	22.0
Sweet whey	10.0
Citric acid	1.0
Iron free vitamin and mineral premix	3.8
Sodium	0.3
Calcium	0.9
Crude oil	14.0
Crude protein	22.0
Crude fibre	0.0
Ash	6.0
Phosphorus	0.7
Lysine	2.1

**Vitamin and mineral mix: (Calculated unites in finished feed)** Vitamin A 24,000 IU/kg; Vitamin D_3_ 5000 IU/kg; Vitamin E 125 IU/kg; Selenium 0.4 mg/kg; Iodine 0.25 mg/kg; Manganese 40 mg/kg; Zinc 50 mg/kg; Copper 135 mg/kg; Vitamin K (menadione) 5mg/kg; Vitamin B_1_ 5mg/kg; Vitamin B_2_ (riboflavin) 5mg/kg; Vitamin B_6_ 5.2 mg/kg; Vitamin B_9_ (folic acid) 1.1mg/kg; Niacin 135 mg/kg; Calcium pantothenate 21 mg/kg; Folic acid 135 mg/kg; Vitamin C 0.15 mg/kg; Biotin300 mg/kg; Betain 114 mg/kg

**Table 2:** Volume of sow milk replacer (SMR) received according to piglet weight

Piglet weight (kg)	Amount of 20% SMR (ml/day)
1.0	50
1.5	80
2.0	130
2.5	190
3.0	240
3.5	290
4.0	350
4.5	420
5.0	520
5.5	630

### Haematological parameters

In addition to weekly onsite blood Hb analysis, a range of blood parameters were quantified by the diagnostic laboratories at the Bristol Veterinary School, Langford, University of Bristol, UK from terminal (age 28 days) blood samples. Briefly, the blood analyses were conducted using a flow cytometry-based analyser (model Advia 2120, Siemens, Germany) which uses laser forward scatter to quantify red blood cell (RBC) and platelets, and also for sizing RBC (mean corpuscular volume, MCV). Haemoglobin (Hb) was measured by lysing the RBCs, oxidising the haem ion to its ferric state, reacting this with 1 H+ ion and 1 H_2_O molecule to form monoaquomonohydroxyferri-porphyrin, which was read at 456 nm. The following calculations were performed:

$$\begin{aligned} {\text{Hematocrit }}\left( {\mathrm{Hct}} \right) = \left( {{\mathrm{RBC}} \times {\mathrm{MCV}}} \right)/10, \end{aligned} $$

$${\text{mean corpuscular Hb}}\left( {{\mathrm{MCH}}} \right) = \left( {\mathrm{Hb/RBC}} \right) \times 10$$

### Metabolic profiling

In order to assess primary metabolite production, plasma samples were analysed using liquid chromatography mass spectrometry (LC–MS). Plasma (50 µL each) was aliquoted into Eppendorf tubes and mixed with 100 µL of acetonitrile (Sigma-Aldrich, UK). This mixture was then centrifuged at ~ 20,000 g for 10 min at 4 °C to facilitate protein precipitation. All samples were then diluted fivefold with 0.1% formic acid. For the determination of primary metabolites, the method of Leung et al.[58] was adopted. This involved separation of primary metabolites in the samples by HPLC (model; i-Series LC-2070, Shimadzu, Japan) using a Discovery HS F5-3 column (2.1 × 150 mm, internal diameter x length; 3 µm particle size; pentafluorophenylpropyl matrix active group; Sigma-Aldrich, USA), with an injection volume of 3 µl, column oven temperature of 40 °C and flow rate of 0.2 mL/min. The target analytes were detected by a triple quadrupole mass spectrometer (Shimadzu, Japan) equipped with LabSolutions software. Peak areas were used to quantify metabolite concentrations to permit comparisons between the treatments (Table 3).

### Gut microbiota DNA extraction and NGS analysis

Stool samples were pre-treated using a modified method from Thiel and Blaut [59] to homogenize and preserve bacterial cells for DNA extraction. Frozen samples (1.6 g) were transferred to sterile 50 mL tubes and diluted in 10 mL of ice-cold 0.05 M PBS buffer (150 mM NaCl, 10 mM Na_2_HPO_4_, 20 mM NaH_2_PO_4_, pH 7.4). The samples were homogenized using sterile glass beads (3 mm diameter; Sigma-Aldrich, USA) by vortexing for 3 min. The homogenized samples were centrifuged at 400 g for 2 min to remove glass beads and faecal debris. The resulting supernatants were transferred to sterile tubes, mixed with three volumes of 4% formaldehyde and incubated on ice for 1 h to preserve the bacterial cells. Following incubation, the samples were centrifuged at 8000 g for 3 min. The cell pellets were resuspended in 4 mL PBS buffer, combined with an equal volume of absolute ethanol and stored at − 20 °C for 20 min. After storage, the pellets were recovered by centrifugation at 8000 g for 3 min and resuspended in 4 ml TE buffer (pH 8.0).

Following pre-treatment, microbial DNA was extracted using a chemical-enzymatic protocol optimized for efficient lysis and the purification of bacterial DNA from complex samples [60]. Aliquots of 500 μL from the pre-treated samples were transferred to sterile 2 mL tubes and treated with 25 μL lysozyme (20 mg/ml) at 37 °C for 30 min. This was followed by the addition of 50 μL of 10% sodium dodecyl sulfate (SDS) and 15 μL of proteinase K (10 mg/mL) in TE buffer (pH 8.0). Samples were then incubated at 55 °C for 2 h. DNA was then purified by gentle mixing with 1 ml of phenol:chloroform (1:1, v/v) and the aqueous phase was collected. The DNA was precipitated with isopropanol, and the precipitate was then washed with 0.5 ml of 70% ethanol, air-dried and finally resuspended in 50 μL of ultra-pure water. Appropriate DNA quality and quantity was confirmed using a NanoDrop ND-1000 spectrophotometer (NanoDrop Technologies, USA). For PCR amplifications, extracted microbial DNA was amplified with universal primers U515F (5’-GTGYCAGCMGCCGCGGTA) and U927R (5’-CCCGYCAATTCMTTTRAGT) for the V4 and V5 regions of the 16S rRNA gene. 16S rRNA gene amplicon-based next-generation sequencing (NGS) was then performed by Novogene (UK) using an Illumina MiSeq instrument to generate paired-end reads and all samples passed their initial quantity and quality controls.

### Bioinformatic and statistical analysis of NGS data

All the microbial bioinformatics analysis was performed using the Reading Academic Computing Cluster (RACC). A total of 2,260,376 16S rRNA gene sequences were obtained from 24 samples, corresponding to an average sequencing depth of 94,182 ± 27,217 reads per sample (mean ± SD). These were processed with an open-source bioinformatics pipeline using DADA2 software in the QIIME2 [61] workflow. ASVs were taxonomically assigned, and for downstream genus-level analyses, ASVs assigned to the same genus were aggregated by summing their abundances to generate genus-level abundance tables. The sequences were pre-processed to remove common contaminants (mitochondrial and chloroplast DNA), amplicon sequence variants (ASVs) that were unassigned at all levels and samples with <2000 reads. This gave a final sample size of 23 (one was excluded from the oral iron treated group due to low read counts) and a total of 1379 ASVs with the summary statistics of sample-wise reads mapped to these ASVs as follows: min 2842; 1st Quartile, 5224; Median, 6642; Mean,:6872; 3rd Quartile, 8864; max, 10,188.

Statistical analyses were performed using R statistical software (v4.4.1, 2024). The vegan package (v 2.7–2) in R [62] was used for alpha and beta diversity determination. For alpha diversity measures, Shannon entropy was used (after rarefying to minimum library size) to measure the balance within the community and Chao1 richness was used to estimate the number of species/features in the abundance table). The aov() function in R was then used to calculate the pair-wise analysis of variance (ANOVA) p-values for alpha diversity. To visualise the beta diversities, principal coordinate analysis (PCoA) (using the cmdscale() function from R’s Vegan package) was used with three different distance measures: (i) Bray–Curtis distance to visualise compositional changes; (ii/iii) Unweighted/Weighted UniFrac distance to estimate, using R’s Phyloseq package (v. 1.48.0) [63], the rooted phylogenetic tree of the ASVs, along with the ASV abundance table, to determine changes between samples in terms of phylogeny. In PCoA plots, ellipses represent the 95% confidence interval of the standard error of groups and were calculated using the ordiellipse() function, again from the Vegan package in R. The top 25 most abundant genera were identified within all treatment groups, and the figures were generated using the ggplot2 package in R.

To report on the dominant elements of the microbiome (the core microbiome), a minimum threshold of 85% prevalence in all samples was used as a cut-off. For this purpose, the microbiome package (v. 1.26.0) in R was used [64]. To identify differential genera (ASVs collated at the genus level using SILVA SSU Ref NR database release v.138), the DESeq2 package (v.1.44.0) was used [65] with the adjusted *p*-value significance cut-off at 0.05 and log2 fold change cut-off of 2. To visualise the resulting taxa, Total Sum Scaling was used followed by a Centralized Log Ratio (TSS+CLR) transformation on the raw abundance values of these genera.

To identify a minimal subset of genera that changed with respect to continuous predictors considered in this study, CODA LASSO regression using the coda glmnet() function from the coda4microbiome package (v. _0.2.4) in R [66] was deployed. The fitted regression was of the form y_i_=β_0_+β_1_logx_1i_+…+β_j_logx_ji_+ϵ_i _(for i-th sample and j-th feature, with x_ji_ being the abundance of feature), and where the outcome y_i_ was a continuous outcome variable. The optimisation method for CODA LASSO regression used a LASSO shrinkage constraint forcing the β-coefficients of the non-significant covariates to zero. The resulting non-zero β-coefficients, by virtue of another constraint was divided into two sets (total sum of these β-coefficients is zero): those that were positively associated with continuous outcome, and those that were negatively associated. Similar to the previous cases, regression at genus level was applied.

### Fluorescence immunohistology

In order to quantify mucosal immune development, frozen serial-Sects. (5 µm) of proximal jejunum, distal jejunum and colon were prepared using a Model OTF cryotome (Brights Instrument Ltd, UK). Sections were air dried for 12 h then fixed by immersion in acetone for 15 min. Slides were then air dried prior to storage at − 20 °C. Non-specific binding sites were blocked using 5% goat and 5% pig serum (Serotech, UK) for 1 h at room temperature and the samples were then stained using the indicated monoclonal antibodies to identify: leucocytes (anti-porcine CD45, clone K252 IE4, ThermoFisher Scientific, UK); monocytes (anti-porcine CD172/SIRPα, clone 74–22–15, Bio-Rad, UK); anti-porcine capillary endothelium (clone MIL11, Bio-Rad, UK); and antigen-presenting molecules (anti-porcine major histocompatibility complex class II DR, clone MSA3, Cambridge Bioscience, UK). Binding was detected with corresponding conjugated secondary antibodies: goat anti-mouse IgG_1_ FITC (ThermoFisher Scientific, UK); goat anti-mouse IgG_2b_ TRITC (Southern Biotech, UK); goat anti-mouse IgE BIOT (Southern Biotech, UK), visualised using AMCA-Avidin D (Vector Laboratories, UK); and goat anti-mouse IgG_2a_ AF633 (ThermoFisher Scientific, UK). To quantify iron-transport protein levels, anti-DMT1/NRAMP2/SLC11A2 (clone 4C6, Novus Biologicals, UK) was used in conjunction with TRITC-conjugated goat anti-mouse IgG_2a_ (ThermoFisher Scientific, UK). Negative controls were prepared where primary monoclonal antibodies were excluded.

Image capture and proportional area of CD45^+^, CD172^+^, MIL11^+^ and MHCII^+^ staining and co-staining, along with divalent metal transporter 1 (DMT1) positive staining, were analysed using an in-house macro in association with ImageJ version 1.53. Briefly, for each piglet and tissue, an Eclipse Ci-S fluorescence microscope (Nikon ECLIPSE C*i*), fitted with appropriate fluorescence narrow-pass filters, was used to generate ten 16-bit grayscale images that were captured by a Nikon Ci-S digital camera. This resulted in 60 representational images for each treatment group per location. Either the lamina propria (LP) or epithelium (EP) were identified as regions of interests (ROI) on the resulting images. An in-house macro was then used to quantify the proportion of pixels in the ROIs corresponding to positive staining, in each of the colour channels and for each area of co-staining (16 in total). The values were logged to achieve normal distributions, as verified by Inman et al*.* [67].

### Statistical analysis

Linear regression was conducted using SPSS (IBM, USA) on the quantitative immunofluorescence data generated using ‘piglet’ as the experimental unit and ‘litter’, ‘location’ and ‘treatment’ as factors. The proportion of pixels positive for CD45^+^, CD172^+^, MIL11^+^, MHCIIDR^+^ and all appropriate combinations were analysed as individual, dependant variables. DMT-1 protein expression was analysed individually using the same experimental unit and factors as above. A value of p <0.05 was significant in the model.

## Results

### Oral and/or IM iron treatment prevented IDA in piglets and raised iron-status-related haematological parameters.

**Fig. 2:**
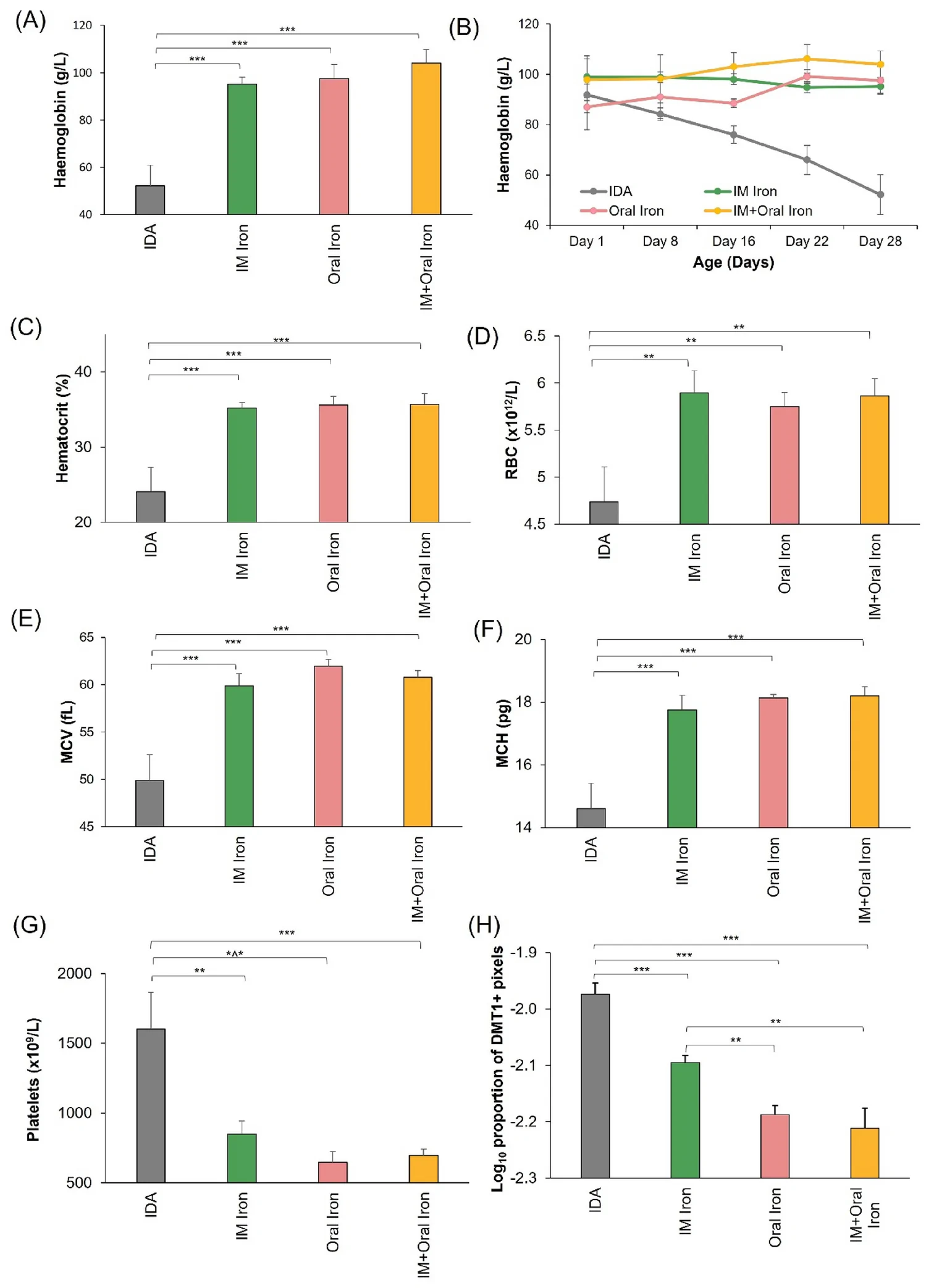
Iron-related parameters were assessed at 28 days of age following either no iron supplementation (IDA) or three different forms of iron treatment: intramuscular injection (IM) (200 mg iron dextran); oral iron sulphate (200 mg FeSO_4_/kg feed); or both (IM and oral iron) from 1 day old. Mean blood haemoglobin (Hb) concentrations (g/L) at day 28 (**A**) and throughout the duration of the trial (**B**) are presented. Haematocrit (**C**)**,** red blood cell (RBC) counts (**D**), mean cell volumes (MCV) (**E**) and mean corpuscular haemoglobin (MCH) values (**F**) are shown together with platelet counts (**G**). The potential for ferrous iron absorption by proximal jejunal enterocytes was explored by quantification of divalent metal transporter 1 (DMT1) expression in 28 day old piglets under differing iron supplementation regimes using fluorescence immunohistology (**H**). Means were obtained using 10 images per piglet. n = 6 litter-matched piglets per treatment group. Error bars indicate SEM; *p < 0.05; **p < 0.01; ***p < 0.001

The concentration of Hb in blood was used as an indicator of iron status [48] and to determine whether the iron treatments had prevented the development of IDA over the course of the trial. By day 28, the three piglet groups that had received iron treatment all displayed mean Hb levels indicating that they were within the normal range (> 90 g/L) [68] and significantly (*p* < 0.001) higher than the mean of the non-iron-treatment group (52.2 g/L) (Fig. 2A) where IDA had developed between day 8 – 16 (Hb concentrations < 80 g/L) (Fig. 2B). Other iron-dependent haematological parameters were also impacted by iron-treatment regimens (Fig. 2C, D, E, and F). The percentage of total blood volume that was made up of red blood cells (haematocrit) was significantly higher (*p* < 0.001) in iron-treated (35.2–35.7%) than non-iron-treated piglets (24.1%) (Fig. 2C). Similarly, red blood cell (RBC) counts (5.86×10^12^–5.74×10^12^/l cf. 4.74×10^12^/L;* p* < 0.001; Fig. 2D) and mean corpuscular volume (MCV) (59.9–62.0 cf. 49.9 fL; *p* < 0.001) were significantly lower in the non-iron treated piglets (Fig. 2E). These results were reflected by the mean corpuscular Hb (MCH) levels which were also significantly (*p* <0.001) reduced in the non-iron-treated piglets compared to iron-treated counterparts (17.8–18.2 cf. 14.6 pg/cell, respectively; Fig. 2F). In contrast, iron treatment significantly reduced blood platelet counts (*p* < 0.001) (6.5×10^11^ to 8.5×10^11^) compared to the non-iron treated piglets (16×10^11^) (Fig. 2G). The results thus indicate that the three alternative iron treatments all successfully prevented IDA and maintained iron-dependent parameters within normal ranges, and further show that there were no significant differences in haematological parameters between the three iron-treatment groups.

### DMT1, responsible for gut-iron absorption, was reduced by oral-iron treatment

To determine whether the iron treatments influenced expression of iron-regulated iron-uptake components in the gut, the levels of DMT1 (ferrous-iron uptake into enterocytes) in the proximal jejunum were determined (at 28 days) by fluorescence immunohistology. Figure 2H shows that the proportion of DMT1 positive pixels was significantly higher in non-iron-treated piglets than in all those treated with iron (*p* < 0.001), indicating that the iron treatments had suppressed DMT1 levels, which is likely the result of an iron-dependent regulatory impact of increased hepcidin levels [69]. In addition, the IM iron only group exhibited a significantly higher level of DMT1 than that of the oral iron groups (with or without IM iron) indicating that oral iron provision caused a greater reduction in DMT1 than IM iron treatment**.**

### Oral-iron supplementation impaired growth

**Fig. 3:**
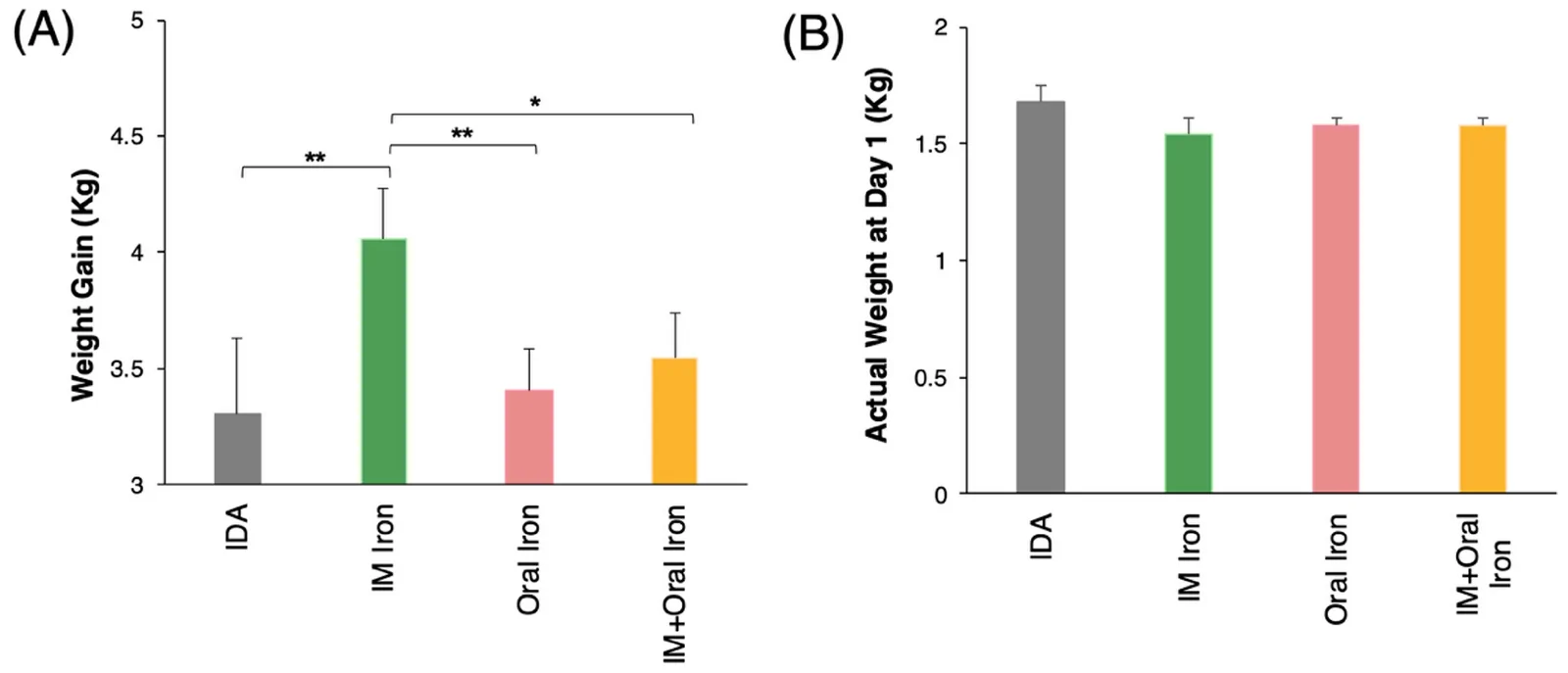
Impact of iron deficiency anaemia (IDA) and different types of iron supplementation on growth (weight gained) in piglets between birth and 28 days old **A**. There were no significant differences in the initial weights of piglets between any of the four treatment groups at 1 day old **B**. Oral iron supplementation (FeSO_4_ at 150 mg/kg) with and without IM supplementation, and IDA were associated with reduced growth rate. n=6 litter-matched piglets/treatment group. Error bars = SEM, **p < 0.01, *p < 0.05

The possibility that the iron regimes applied might differentially impact growth was tested by monitoring body weight over the course of the trial. At 28 days old (end of trial), mean weight gain in the oral iron supplemented piglets (3.40–3.54 kg) was significantly lower (*p* < 0.05) than in IM iron only treated siblings (4.05 kg). The non-iron treated (anaemic) piglets also displayed a significantly lower weight (*p* <0.01, 3.30 kg) at age 28 days compared to the piglets receiving IM iron only. Thus, the piglets that only received IM iron exhibited a considerably and significantly greater weight gain (of 0.51–0.75 kg; 14–19%) than observed for the other three piglet groups (Fig. 3A). The results therefore suggest that although oral iron supplementation (at the dose provided) prevents IDA, in contrast to IM iron treatment, it also supresses piglet growth. It should be noted that there were no significant differences in starting weights between any of the treatment groups (Fig. 3B).

### IDA resulted in reduced levels of metabolites related to amino acid and energy metabolism

Since the iron regimes applied influenced both anaemia status and growth, a possible impact of iron treatment on metabolism was explored by LC–MS analysis of key plasma metabolites at day 28 (Table 3). This revealed multiple significant reductions in metabolite levels within the anaemic piglets. The greatest effect was a 13.9- to 25-fold lower level of creatine in the anaemic (non-iron treated) piglets, with respect to those measured in the iron-treated piglets (Table 3). Creatinine, a derivative of creatine, was also significantly reduced in the anaemic piglets (by 2–5- to 4.5-fold). These low levels could be indicative of low muscle mass and/or malnutrition in the iron-deprived piglets [70]. The anaemic piglets also displayed reduced levels of plasma amino acids; of the 20 that were detectable, 11 (including arginine, as highlighted below) displayed significantly reduced levels (from 2.2- to 5-fold; mean of 3.7-fold) in the anaemic piglets relative to iron-treated piglets (Table 3). The amino acid dimethylglycine was also significantly (4.0-fold) lower in the anaemic piglets. These low amino acids levels during anaemia may contribute to the reduced creatine levels (and a reduced muscle mass, as suggested above). In addition, three TCA cycle intermediates were significantly lower in the anaemic (non-iron treated) piglets with respect to levels in the iron-treated groups (Table 3): mean aconitic acid, citric acid and 2-ketoglutaric acid levels were 11.4-, 3.8- and 2.3-fold lower (*p* < 0.001, < 0.05 and < 0.05), respectively (Table 3). However, there were no significant changes for three other detectable TCA cycle metabolites (fumarate, isocitrate and succinate, data not shown). The reduced levels of TCA cycle intermediates are suggestive of supressed energy production and amino acid biosynthesis activity, which might explain the lower levels of plasma amino acids. The anaemic piglets also displayed significantly lower levels of two urea cycle intermediates: 9.9-fold lower citrulline (*p* < 0.001) and 5.0-fold lower arginine (*p* < 0.001), although ornithine and argininosuccinate (data not shown) were not affected. This may be related to a reduced need to dispose of ammonia due to the observed lower amino acid levels.

**Table 3:** Impact of iron deficiency anaemia IDA and different forms of iron supplementation on host systemic metabolite production in 28-day old piglets

Associated pathways	Metabolite	Concentration (peak area) ± SE			
		Anaemic piglets (No Iron)	IM iron injection (200 mgs)	Oral iron (150mgs/kg feed iron sulphate)	IM & oral iron
Tricarboxylic acid (TCA) cycle	Citric acid	2,273,121±1,135,143	9,791,337±2,055,168^a**^	9,392,108±1,331,781^b**^	7,149,385±719,089^c*^
	Aconitic acid	50,071±6046	543,363±166,088^a**^	570,280±125,155^b**^	595,522±89,876^c**^
	2-ketoglutaric acid	9135±1741	18,474±5349.97^a^	20,419±2717^b*^	25,958±2624^c**^
Urea cycle	citrulline	460,191±370,484	4,517,798±1,283,756^a***^	4,109,902±470,667^b**^	5,044,502±353,760^c**^
	Arginine	7,414,743±3,443,185	39,962,874±9,607,885^a**^	40,772,509±8,588,724^b**^	30,730,701±6,383,845^c*^
Non-essential amino acids	Methionine	23,953,069±18,171,183	91,075,625±19,483,786^a*^	117,515,427±14,901,057^b***^	83,163,361±13,556,616^c*^
	Serine	1,596,615±949,420	4,910,191±1,048,132^a**^	5,830,155±357,290^b***^	5,760,003±275,040^c***^
	Asparagine	526,431±307,115	1,587,457±355,684^a**^	1,815,804±221,888^b***^	1,551,369±276,874^c**^
	Glycine	800,939±496,659	2,731,429±595,255^a**^	3,129,219±385,656^b***^	3,999,213±496,744^c**^
	Glutamine	13,825,651±8,214,481	44,105,411±11,034,450^a**^	47,043,060±9,065,262^b***^	56,055,431±5,153,707^c***^
	Glutamic acid	2,224,961±1,337,819	6,411,075±1,853,800^a^	11,197,229±2,939,089^b**^	12,426,045±2,702,284^c***^
	Proline	13,063,259±7,427,003	25,983,666±5,114,158^a^	30,273,174±2,132,942^b**^	30,807,151±3,320,438^c**^
Essential amino acids	Leucin	115,519,681±97,178,922	378,636,058±79,954,476^a**^	492,505,521±44,100,219^b***^	392,798,874±64,522,240^c**^
	Iso-leucin	113,245,800±95,187,722	373,097,738±78,475,535^a**^	485,993,853±43,069,709^b***^	389,988,390±63,488,957^c**^
	Cystine	76,264±39,412	214,930±67,205^a*^	277,597±30,751^b***^	239,814±26,515^c**^
Other metabolites	Creatine	1,776,609±445,424	24,868,218±7,013,743^a*^	37,643,603±11,998,774^b***^	45,327,878±9,229,647^c***^
	Creatinine	5,690,790±4,556,983	20,221,504±3,875,653^a**^	25,454,400±1,814,985^b***^	23,130,368±1,729,749^c***^
	Dimethylglycine	60,461±39,981	181,039±42,729^a**^	277,665±27,547^b***^	264,395±20,798^c***^
	Acetyl-carnitine	7,488,586±3,688,499	31,464,106±5,489,625^a**^	45,418,631±14,472,275^b***^	37,648,356±7,812,154^c***^
	Lactic acid	3,362,735±2,080,952	9,379,576±2,259,844^a**^	11,464,058±1,199,622^b***^	11,589,747±655,656^c***^

n = 6 litter-matched piglets per treatment group. *p < 0.05; **p < 0.01; ***p < 0.001.Significance indicates differences compared to piglets with IDA, no significant differences were identified in blood metabolites between the piglets which received iron supplementation (oral, IM and oral+IM)

Acetylcarnitine was also significantly (5.1-fold) lower in the anaemic piglets than in the iron-treated piglets (Table 3). This compound has a role in fatty acid metabolism and energy generation, and low levels are associated with malnutrition [71]. Lactic acid concentrations were also significantly lower (3.2-fold) in the anaemic piglets, compared to the iron-treated piglets. Although under normal circumstances IDA is usually associated with increased blood lactic acid related to reduced oxygen delivery to muscle, our piglets were severely anaemic which induced lethargy; severely limiting exercise has previously been shown to reduce overall lactate accumulation in IDA [72].

Taken together, these results show that IDA was associated with reductions in a range of metabolites associated with amino acid and energy metabolism. The data also indicate that all three iron treatments rescued the reduced metabolite production observed in the anaemic piglets, to similar degrees. Indeed, there were no significant differences in any of the detected TCA or urea cycle intermediates, amino acids, creatine, lactic acid or acetylcarnitine between the piglet groups receiving the three different types of iron treatment. Thus, the effects indicated above appear to be related to IDA, not growth. This indicates that the reduced weight gain, as induced by oral iron supplementation with respect to IM treatment, was not related to the reductions in energy and amino acid metabolism that were associated with IDA.

### Effects of iron supplementation on microbial diversity and composition

#### Alpha and beta diversity

**Fig. 4:**
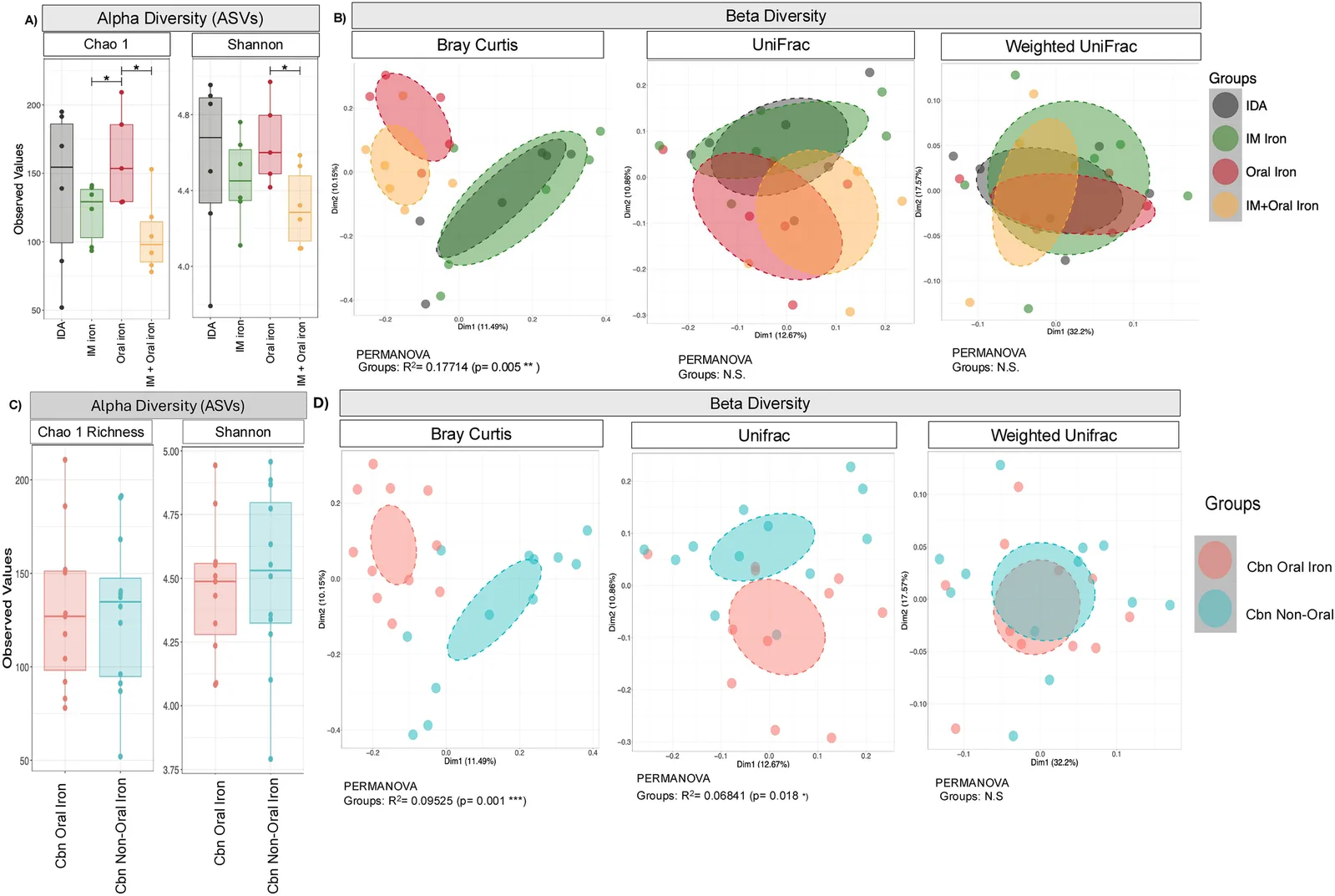
*Impact of iron-treatment on the alpha and beta diversity.* (**A** and **C**) Alpha diversity (Chao 1 Richness and *Shannon* entropy) comparison of bacterial ASVs. (**B** and **D**) Beta diversity represented by principal coordinate analysis (PCoA) plots with each axis showing the percentage variability explained by that axis. Ellipses represent 95% confidence interval of the standard error for a given group. Three different distance measures were deployed: *Bray–Curtis distance*, *Unweighted UniFrac* and *Weighted UniFrac*. PERMANOVA statistics using these distance measures are shown beneath the PCoA plots to indicate significant differences between the groups with the R^2^ value showing percentage variability

For the gut bacterial populations, significant reductions in alpha diversity for both richness (Chao1) and diversity (Shannon index) were detected in the IM+Oral Iron group compared to the IDA group (*p* < 0.05 and *p* < 0.01, respectively), indicating that the combined iron treatment negatively impacted overall microbial diversity (Fig. 4A). Also, the two IM treatment groups showed lower diversity than the non-IM treatment groups. The IM Iron and Oral Iron groups did not show significant differences from the IDA group, suggesting that the combined iron treatment may have resulted in a synergistic effect in the reduction of alpha diversity.

Beta diversity analysis also demonstrated compositional shifts in the gut microbiota in response to iron treatment (Fig. 4B). Bray–Curtis’s dissimilarity revealed significant differences among all groups (R^2^ = 0.17714, *p* = 0.005), suggesting that iron treatment alters overall microbial community structure. However, no significant differences were observed by UniFrac, indicating that phylogenetic relationships among taxa were not substantially affected.

The difference detected by Bray–Curtis analysis largely relates to the distinct clustering of the two oral-iron groups relative to the two non-oral iron groups. Indeed, when the piglets were combined into just two groups on the basis of oral iron provision (group 1—combined oral Iron, i.e. Oral Iron and Oral Iron +IM Iron; and group 2—combined non-oral iron, i.e. IM Iron + IDA), a clear and significant separation of the two groups was apparent by both Bray–Curtis (*p* = 0.001***) and Unifrac (*p* = 0.018*) (Fig. 4D). However, no significant differences were found in alpha diversity analysis of these two groups (Fig. 4C). Thus, oral iron supplementation had a significant impact on the composition of the piglet gut microbiota population whereas IM treatment had no significant impact with respect to the non-treatment group. A pairwise beta diversity comparison of all four treatment groups is given in Table S1.

### Comparison of the effects of the iron treatments on the 25 most abundant bacterial genera and core microbiome

**Fig. 5:**
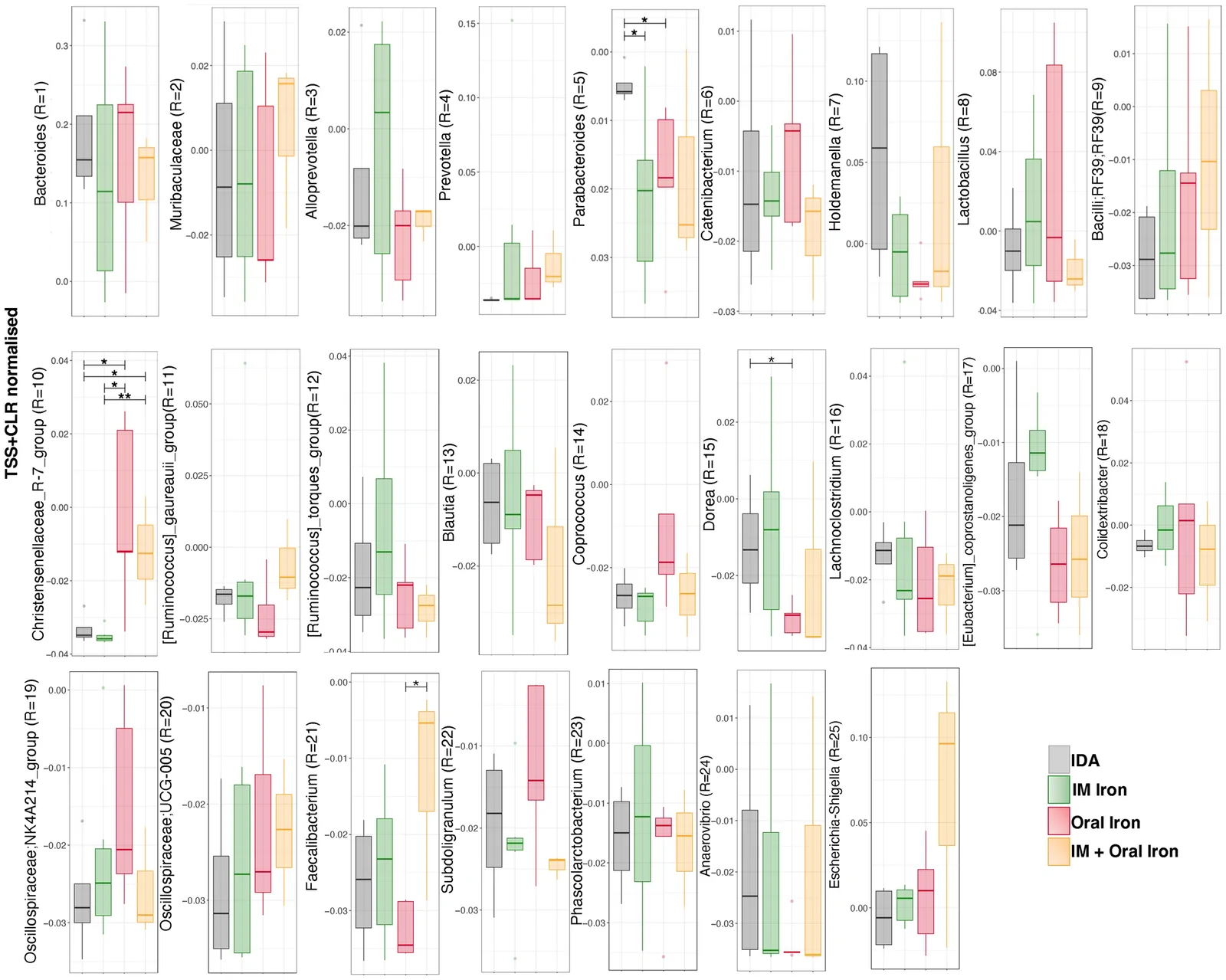
The TSS + CLR normalized expressions of the top 25 most abundant genera observed n the piglet gut microbiota iron-treatment dataset. Significant differences in abundance between treatments are indicated in the corresponding boxplots as determined ANOVA: *p < 0.05; **p < 0.01; ***p < 0.001

**Fig. 6:**
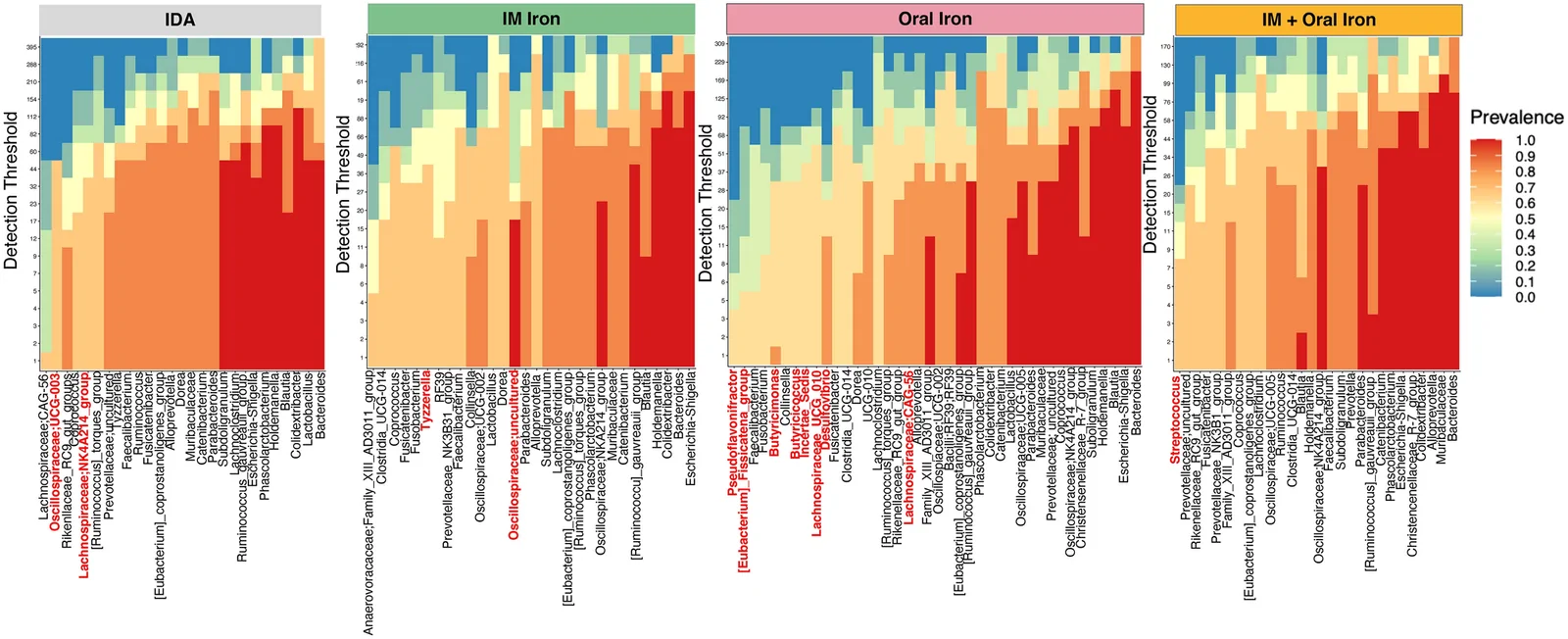
The core gut-microbiome heatmaps for all treatment groups representing the genera achieving a minimum of 85% prevalence across all 23 piglets. The y-axis represents the detection thresholds from lower to higher abundance values and color shading shows the prevalence of each taxon among samples for each abundance threshold. The prevalence decreases as the detection threshold increases. Those genera unique to each group are indicated in red text

The top 25 most abundant genera across treatment groups, presented in abundance-ranking order (R1-R25), are shown in Fig. 5. While dominant taxa such as *Bacteroides* (R1) and *Muribaculaceae* (R2) remained relatively consistent across treatment groups, several taxa showed significant variation. *Christensenellaceae_R-7_group* (R10) was significantly more abundant in the Oral Iron group compared to all other groups (*p* < 0.05 to < 0.01). *Parabacteroides* (R5) showed higher abundance in the Oral Iron group compared to the IM+Oral Iron group (*p* < 0.05), while *Lachnoclostridium* (R16) was significantly increased in the IM Iron group compared to IM+Oral Iron (*p* < 0.05). Additionally, *Faecalibacterium* (R21) was more abundant in the IM+Oral Iron group than in the IDA group (*p* < 0.05). These findings indicate that certain taxa are selectively influenced by iron treatment and by the manner in which the iron treatment is delivered. This effect was further explored by core microbiome analysis (Fig. 6) which considered those bacterial genera that persisted in ≥85% of samples in all four treatment groups. In this way, 31 bacterial genera were found to be shared within the core gut microbiomes of all the piglets, irrespective of iron-treatment group. These shared genera included *Bacteroides*, *Colidextribacter*, *Holdemanella*, *Blautia* and *Escherichia-Shigella* (Fig. S1). However, several unique genera associated with specific treatment groups were also identified: *Oscillospiraceae*(UCG-003*)* and *Lachnospiraceae*(NK4A214_group) in the IDA group; *Tyzzerella* and *Oscillospiraceae*(uncultured) in the IM Iron group; *Pseudoflavonifractor, Butyricimonas*, *Butyricicoccus*, *Desulfovibrio*, *Lachnospiraceae_UCG_010* A and *Lachnospiraceae*(CAG-56) in the Oral Iron group; and *Streptococcus* in the IM+Oral Iron group. These iron-treatment associated differences are a further indication of how the iron regime applied impacts the piglet gut microbiota.

### Differential abundance and correlation analysis

Given the significant difference in beta-diversity between the combined oral and non-oral iron groups (Fig. 4D), as well as the significant oral iron-treatment related impact on piglet weight gain (Fig. 3), the data were further analysed to determine which bacterial genera might contribute to these effects. Thus, two approaches were deployed: DeSeq2 analysis was used to discover genera displaying a significant >2 log2 fold difference in response to oral iron treatment; and correlation analysis using a regression model (CODA-LASSO) was employed to identify genera positively or negatively associated with piglet total-weight gain in response to iron treatment.

**Fig. 7:**
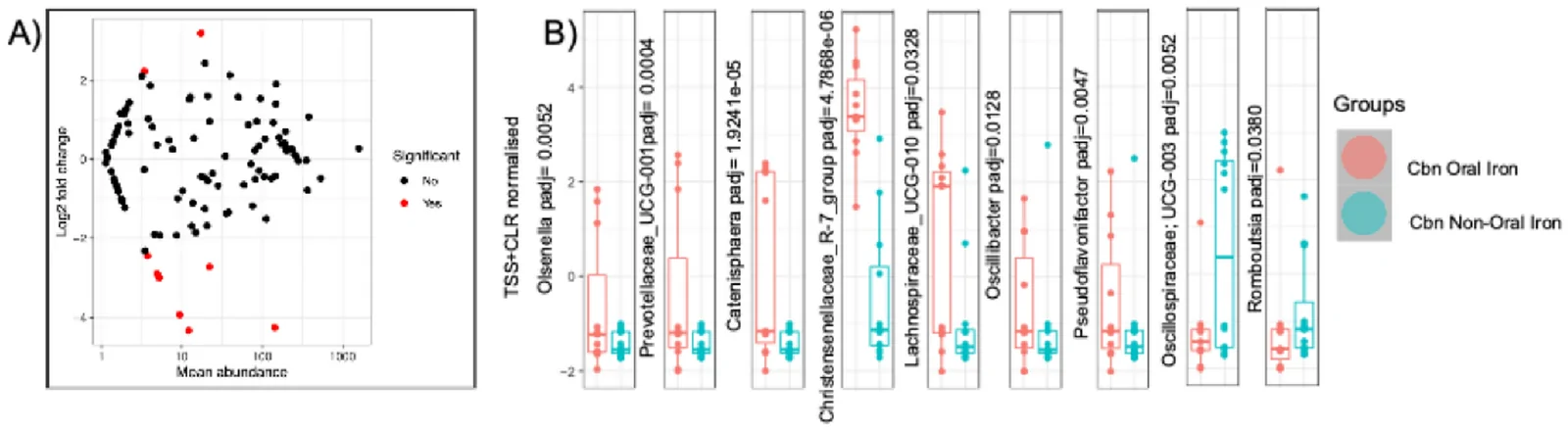
*Differential abundance analysis comparing the Combined Oral Iron and Combined Non-Oral Iron treatment groups using DESeq2*. (**A**) Mean abundance (MA) plot for bacterial genera. Genera significantly (> 2 log2-fold) enriched or depleted by oral-iron are represented as red dots. (**B**) Expression plot (TSS+CLR normalised) of individual genera found to be significantly enriched (red bars) or depleted (green bars) by oral iron (adjusted *p*-value significance cut-off of 0.05; log2 fold change cut-off of 2)

The DeSeq analysis (Fig. 7) revealed that oral iron significantly enhanced the abundances of seven genera (~ 5–20-fold: *Olsenella*, *Prevotellaeae* UGC-001, *Catenisphera*, *Christensenellaceae* R-7, *Lachnospiraceae* UCG-010, *Oscillibacter* and *Pseudoflavonifactor*) and significantly supressed two genera (~ 5–9-fold: *Oscillospiraceae* UCG-003 and *Romboustia*). Of these, the most notable effects were the oral-iron enhancement of *Christensenellaceae* R-7 group (19-fold) and *Lachnospiraceae* UCG-010 (6.6-fold), and the oral-iron suppression of *Oscillospiraceae* UCG-003 (9 fold). The abundance of the *Christensenellaceae* has previously been reported to be inversely associated with BMI [73]. Such an association is supported here as oral-iron treatment both enhanced the *Christensenellaceae* (Fig. 7B) and suppressed weight gain (Fig. 3).

**Fig. 8:**
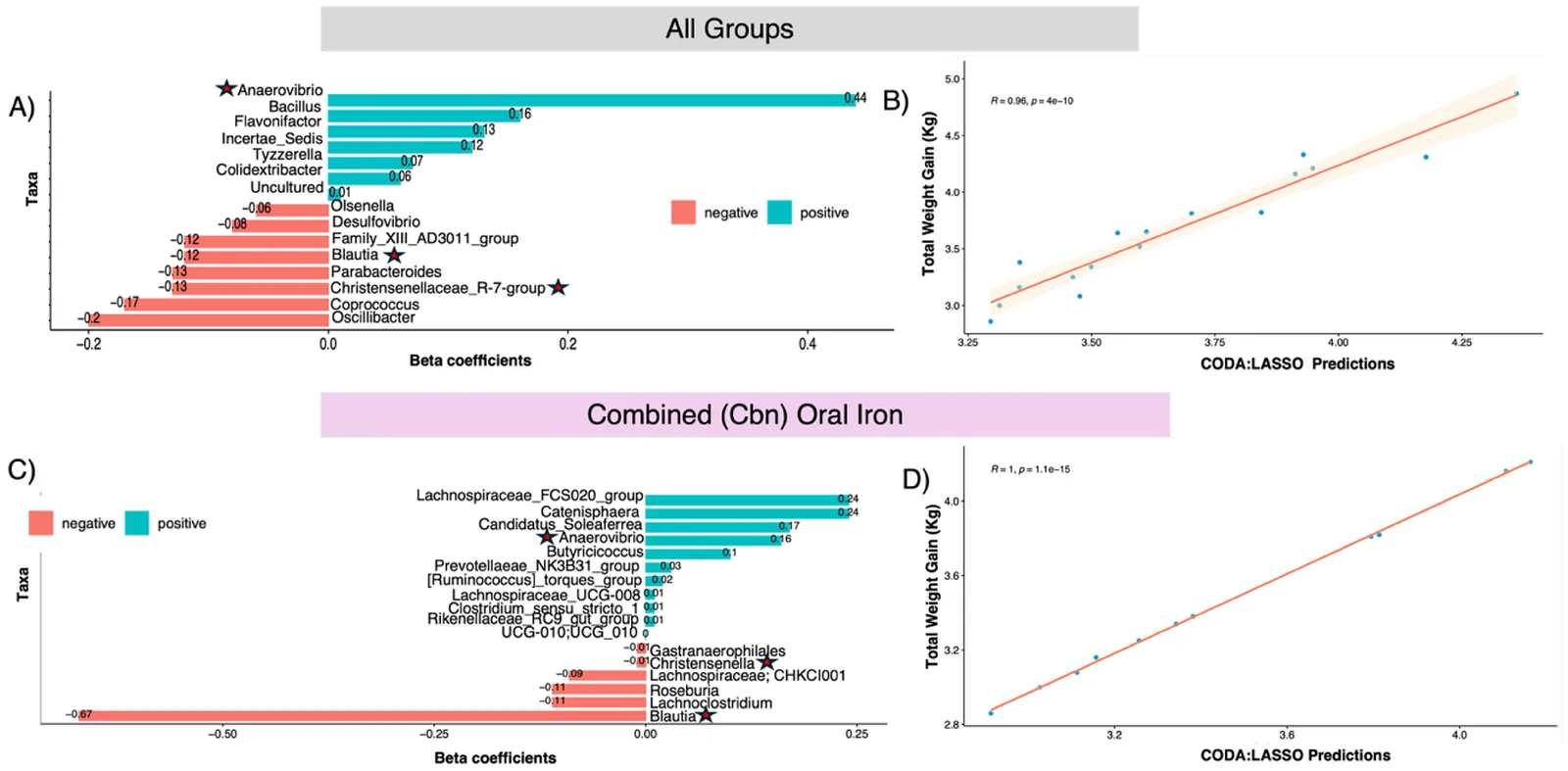
Identification of piglet gut microbiota genera positively or negatively associated with piglet weight gain. Microbial taxa signatures were generated using CODA-LASSO to identify signatures with maximum discrimination accuracy between all treatment groups (**A**) and in the Combined Oral Iron group (**C**). The signatures were defined by the relative abundances of two groups of taxa where one group is composed of taxa with positive coefficients (green) (with respect to weight gain) in the regression model and the other group is composed of taxa with negative coefficients (red). Taxa shared between **A** and **C** are indicated by stars. The bar graphs represent the taxa signatures and their coefficients. The scatter plots show the prediction for classification accuracy i.e. relationship/correlation between all treatment groups (**B**) and the Combined Oral Iron group (**D**)

Total weight gain in piglets over the trial period was correlated with bacterial abundance (at genus level) using a regression model (CODA-LASSO) in order to identify taxa that were associated with total weight gain across all four treatment regimes (Fig. 8). In this way, two distinct sets of gut-microbiota taxa were identified, one where abundance increased across treatment groups as piglets gained weight, and another where abundance decreased with weight gain (Fig. 8A and B). Specifically, weight gain in piglets was associated with an increase in abundance for six genera (*Anaerovibrio, Bacillus, Flavonifactor, Incertae Sedis*, *Tyzzerella* and *Colidextribacter*) and decreases for eight genera (*Oscillobacte*r, *Desulfovibrio*, Family XIII AV3011, *Blautia*, *Parabacteroides*, *Christensenellaceae* R-7, *Coprococcus* and Olsenella) (Fig. 8A). To explore the relationship between the gut microbiota and weight gain in response to oral iron treatment, CODA-LASSO was used to identify the genera associated with a change in abundance with weight gain for the 12 piglets that received combined oral iron supplementation (Fig. 8C and D), since this group displayed a significantly slower weight gain (Fig. 3). Thus, 11 genera were positively correlated with total weight gain, while the abundances of 6 genera were negatively correlated (Fig. 8C). Interestingly, three of the weight-gain-associated genera (*Anaerovibrio, Blautia* and *Christensenella*) were common between both groups (the ‘All’ iron group, Fig. 8A; and the ‘Combined Oral Iron’ group, Fig. 8C) suggestive of a relatively strong weight-gain association for these genera*.* However, the model did not identify any genera associated with weight gain in the ‘Combined Non-Oral Iron’ group suggesting no significant associations with weight gain in the absence of oral iron.

To determine whether bacterial taxa altered by oral iron supplementation were also associated with growth, the differentially abundant taxa identified by DESeq2 were compared with taxa selected by the CoDA LASSO model. Catenisphaera was identified by both analyses, being significantly enriched in the oral iron group and positively associated with total weight gain. In addition, members of the *Christensenellaceae,* *Lachnospiraceae,* and *Prevotellaceae* families were represented in both analyses, although the specific genera differed. Specifically, oral iron supplementation enriched *Christensenellaceae*_R-7_group**,** while *Christensenella* was negatively associated with total weight gain in the CoDA LASSO model, indicating concordance at the family level. Similarly, oral iron supplementation enriched *Lachnospiraceae*_UCG-010 and *Prevotellaceae*_UCG-001, whereas other members of the *Lachnospiraceae* and *Prevotellaceae* families were identified as growth-associated taxa in the CoDA LASSO analysis.

### IDA and iron supplementation impact immune-associated protein expression along the pre-weaned piglet gut

**Fig. 9:**
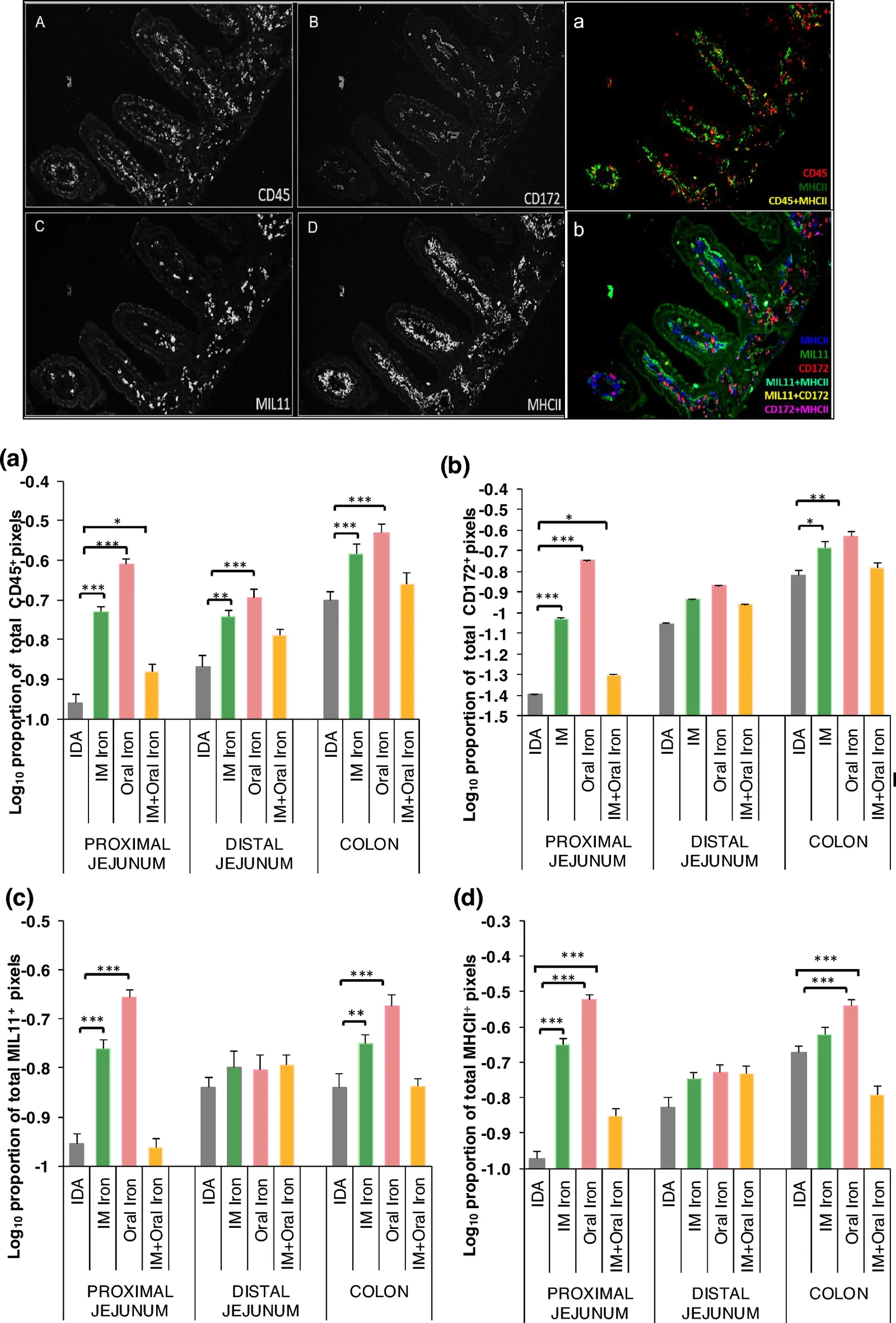
Quantitative fluorescence immunohistology was used to explore immune development in the lamina propria (LP) in response to iron deficiency anaemia (IDA) and iron supplementation (intramuscular injection (IM) (200mgs iron dextran), oral iron sulphate (200 mgs FeSO_4_/kg feed) or both (IM and oral iron) in 28 day old piglets at different locations along the intestinal tract. Representative images of CD45^+^ (**A**), CD172^+^ (**B**), MIL11^+^ (**C**) and MHCII (**D**) stained section of proximal jejunum in a healthy piglet (IM iron treated). Each image is the same field of view. Merged images show CD45^+^ (red), MHCII^+^ (green) and co-expression of CD45^+^MHCII^+^ (yellow) (**E**)**.** MHCII^+^ (blue), MIL11^+^ (green) and CD172^+^ (red) and co-expression by MIL11^+^ and MHCII^+^ (cyan), MIL11^+^ and CD172^+^ (yellow) and CD172^+^ and MHCII^+^ (magenta) **(F)**. Log_10_ proportion of CD45^+^ (**G**), CD172^+^ (**H**), MIL11^+^ (capillary endothelium) (**I**) and MHCIIDR^+^ (**J**) pixels in the different tissue locations are shown, Means were obtained using 10 images per piglet. IM = intramuscular injection of iron (200 mgs iron dextran). Error bars = SEM, n = 6 litter-matched piglets/treatment group. *p < 0.05; **p < 0.01; ***p < 0.001

Fluorescence immunohistology was used to quantify the expression of immune-associated proteins along the intestinal tract in response to IDA and the different iron treatments in pre-weaned piglets following 27 days of nutritional intervention (from 1 day old). Representational images of CD45 (Fig. 9A), CD172 (B), capillary endothelium (MIL11, C) and MHCII (D) show the same field of view of the distal jejunum in a representative healthy piglet. The images indicate that CD45^+^ cells were abundant and widely distributed throughout both the lamina propria (LP) and epithelium, while CD172^+^ cells, CE^+^ and MHCII^+^ molecules were localized and clustered only in the LP. Figure 9E shows the expression of CD45^+^ (red), MHCII^+^ (green) and CD45^+^MHCII^+^ co-expression in yellow. Combined image (Fig. 9F) show MHCII^+^ (blue), MIL11^+^ (green) and CD172^+^ (red) staining, co-expression of MIL11^+^MHCII^+^ (cyan), CD172^+^MIL11^+^ (yellow) and CD172^+^MHCII^+^ (magenta). The results indicate that IDA was associated with significant reductions in the expression of CD45 (Fig. 9G), CD172 (H), MIL11 (I) and MHCII (J) compared to piglets which received IM iron, especially in the proximal jejunum (*p* < 0.01 for all). This was also the case in colonic tissue for all immune-associated targets (*p* < 0.05), except for MHCII^+^ where the difference was not significant. Interestingly, colonic and proximal jejunal expression of all quantified immune-associated proteins was highest in the piglets that received oral iron only (*p* < 0.01) compared to the IDA piglets, whereas this was reversed in piglets which received both IM and oral iron, where expression levels were often not significantly different to those expressed in the IDA piglets (Fig. 9G, H, I, J). The piglets that received IM iron only could be considered to act as controls since this treatment best reflects the normal situation for both human infants and piglets. This is because they benefit from an iron endowment from their mothers before birth and are exposed to very limited gut iron from maternal milk. In these ‘control’ piglets, expression of both CD45 and CD172 increased along the intestinal tract, while MIL11 and MHCII expression remained similar throughout.

### IDA and iron supplementation affect the potential for antigen presentation in the gut mucosa

**Fig. 10:**
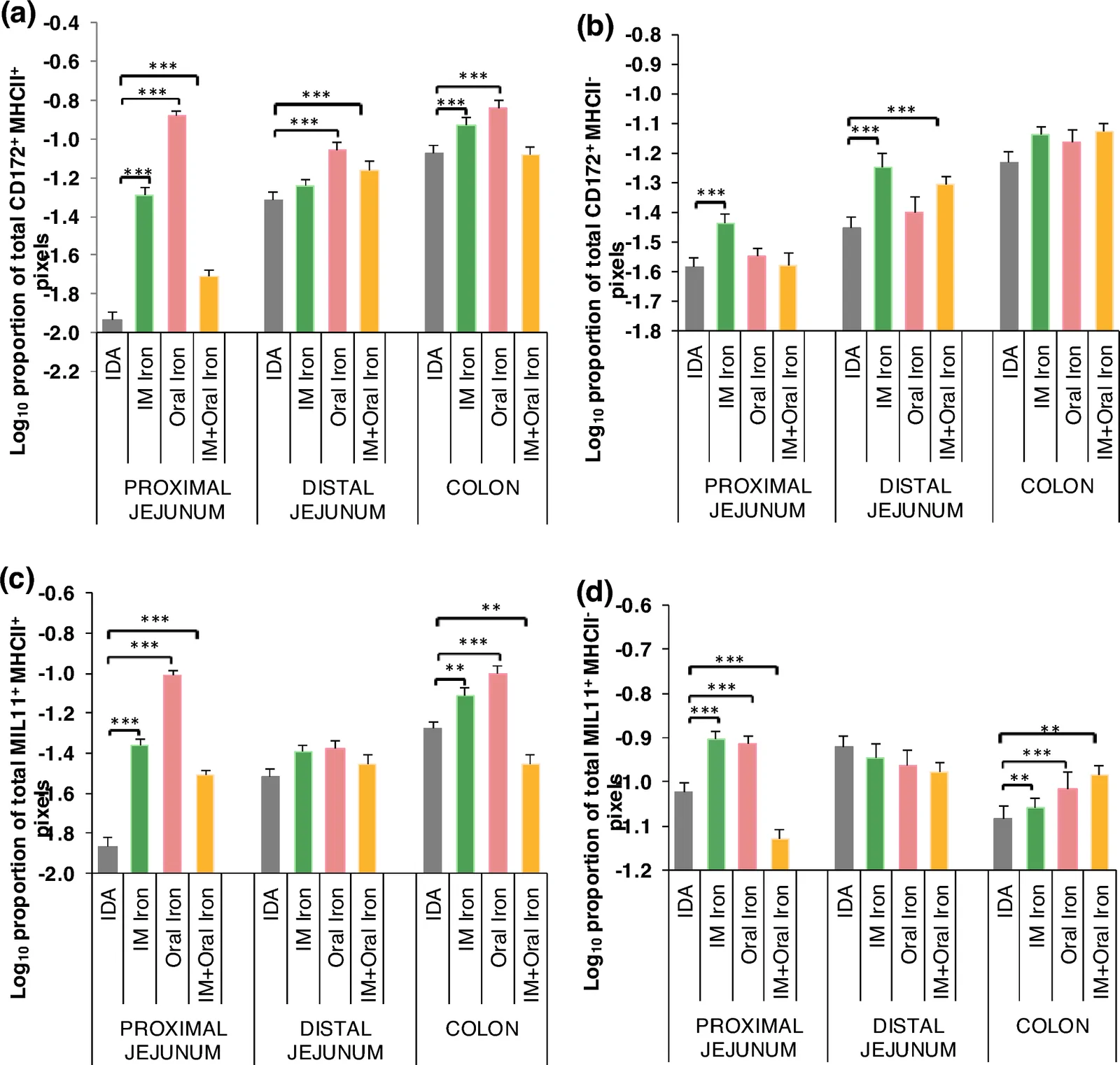
The potential for antigen presentation in the lamina propria (LP) along the intestinal tract in response to iron deficiency anaemia (IDA) and different forms of iron treatments; oral (200 mgs FeSO_4_/kg feed with or without an intramuscular (IM) injection (200mgs iron dextran at 1 day old were explored in 28-day old piglets using quantitative immunohistology. CD172^+^ staining with (**a**) and without (**b**) co-expression with MHCII, along with MIL11^+^ with (**c**) and without (**d**) MHCII co-expression are presented. Means were obtained using 10 images per piglet. Error bars = SEM, n = 6 litter-matched piglets/treatment group. *p < 0.05; **p < 0.01; ***p < 0.001

Since antigen presentation initiates immune responses and is especially important during early-life when the immune system is rapidly developing appropriate responses to harmless and potentially dangerous antigens, we explored aspects of antigen presentation. Following quantification of positive staining for CD172, CE (MIL11) and MHCII co-expression, combinations were analysed to give an indication of the potential for antigen presentation by monocytes and CE in the LP along the intestinal tract, in response to the different treatments. IDA was associated with significant reductions in CD172 in both the presence (Fig. 10A) and absence (B) of MHCII, compared to iron-treated piglets at all three locations assessed (proximal and distal jejunum, and colonic tissue). This is consistent with not only a reduced number of monocytes, but also reductions in the potential for these monocytes to present antigen and initiate immune responses. All iron treatments not only increased the proportion of CD172 staining, but also significantly increased the amount of CD172 and MHCII co-staining, suggesting that iron supplementation restored potential for antigen presentation along the intestinal tract, largely via dendritic cells and macrophages. However, the extent of this restoration was highly dependent on location; CD172^+^MHCII^+^ co-staining was higher in the distal jejunum and colon compared to the proximal jejunum, although this was dependent to a degree on which type of iron supplementation was used, and whether piglets had IDA.

Stromal cells have been demonstrated to present antigens outside of lymph nodes in piglet intestines and our results show that the potential for CE cells to present antigen (MIL11^+^ and MHCII^+^ co-staining) was impacted by both treatment and location along the intestinal tract (Fig. 10C) indicating that treatments did not have uniform effects throughout the gut. Interestingly, oral iron only resulted in significant increases in MIL11^+^MHCII^+^ co-staining in the proximal jejunum and colon, but not in the distal jejunum. For piglets receiving oral iron with or without IM treatment, the potential for CE cells to present antigen was significantly reduced in the proximal jejunum and colon, but not in the distal jejunum. As with CD172^+^MHCII^+^ co-staining, MIL^+^MHCII^+^ expression was most affected by iron status in the proximal jejunum compared to the distal jejunum and colon. This was also true for MIL11 expression in the absence of MHCII co-expression (Fig. 10D), where the most significant differences in response to treatment (iron or IDA) were observed in the proximal jejunum compared to the distal jejunum and colon. However, neither IDA nor any of the iron treatments affected the expression of MIL11 in the absence of MHCII in colonic LP. The overall model showed that there were significant interactions between ‘treatment’ and ‘location’ along the intestinal tract (*p* < 0.001).

## Discussion

Due to its key role in several fundamental biological processes, iron is an essential micronutrient required by all animals, including humans. However, iron deficiency anaemia (IDA) is a substantial world-wide human health problem, especially for infants, and iron supplementation is commonly used to address this issue. Here we assessed the impact of IDA, intra-muscular (IM), oral and IM+oral (combined) iron treatments on growth, iron status, host metabolism, gut microbiota composition and immune development along the intestinal tract using piglets as models for human infants. The results show that, as expected, a lack of iron treatment caused a significant reduction in growth rates (a stunting effect), and that oral or IM iron, and combination of oral and IM iron treatment, were all sufficient in preventing the development of IDA. In addition, there were no significant differences in any of the measured iron-dependent blood parameters, or in host plasma metabolite production, resulting from the different forms of iron treatment applied. However, the expression of the duodenal iron transporter, DMT-1, increased in response to IDA while oral iron (with or without IM iron) was associated with decreased expression of DMT1 in the proximal jejunum. This response of DMT1 to oral iron matches that reported previously [74]. Importantly and surprisingly, the results also show that administration of oral iron (with or without IM iron) caused significant reductions in weight gain (resulting in stunting) compared to piglets receiving IM iron treatment only. Indeed, the weight of piglets receiving oral iron was comparable to that of piglets that received no iron treatment and thus rapidly developed IDA.

Since measured host parameters remained largely constant in response to the different forms of iron treatment, despite significant reductions in growth rate for piglets receiving oral iron, the impact of iron treatment regimes on the composition of the gut microbiota was explored by 16S rRNA amplicon NGS analysis. Beta-diversity analysis showed that oral iron treatment caused a significant shift in gut microbiota composition such that distinct clustering was observed between the oral-iron group with respect to the non-oral iron groups. Distinct bacterial taxa were found to be associated with the reduced growth rate in piglets that received oral iron. As the gut microbiota is the primary driver of immune development during the neonatal period, the effects of the iron-treatment-related changes in gut bacterial populations upon the immune development of the gut were explored. We found significantly lower immune-associated protein expression in IDA piglets compared to iron supplemented siblings, and the effects of both IDA and iron supplementation varied along the intestinal tract. In addition, IDA and iron treatment affected the absolute expression of CD45, CD172, MIL11 (capillary endothelium) and MHCII in the LP, and impacted the potential for antigen presentation by both professional (CD172^+^) and stromal (CE) antigen presenting cells in the gut mucosa, which again was highly location dependent.

Growth rate is a useful proxy measure of general health in young animals [75]. Our data show that the type of iron used to prevent the development of IDA requires careful consideration. Whilst there are negative implications associated with IM iron injections, including iron overload and occasional inflammation/infection at the injection site [76], our results demonstrate that the alternative form of iron treatment, oral iron, drives significant reductions in piglet growth rate, even in the presence of additional IM iron. Importantly, infants can receive iron fortified formula in addition to oral iron supplementation, and this may be particularly relevant for low-birth-weight infants receiving subsequent inadequate nutrition. This is also highly relevant to the pig industry where the feed for young piglets is fortified with high concentrations of iron, often in excess of 250 mg/kg of dry feed. Although piglets are particularly susceptible to developing IDA during the neonatal stage for the reasons outlined in the introduction, they routinely receive an injection of iron dextran (200 mg) during the first days of life. For this reason, they may not require additional dietary iron in early life, which could cause them to take longer to reach market weight, thus contributing to production costs in an industry that has tight profit margin.

The levels of blood metabolites in anaemic piglets differed significantly from those in piglets which received iron supplementation and thus avoided developing IDA. This was expected and has previously been reported in post-weaned piglets receiving low iron diets (65 mg/kg dry feed) with no initial IM injection of iron dextran, where reduced weight-gain also presented [77]. Metabolites associated with the TCA cycle (citric acid, aconitic acid, and 2-ketoglutaric acid), urea cycle (citrulline and arginine), methylation (methionine) and phosphorylation (serine) processes were significantly reduced. This is consistent with a study in human cell lines (K-562) [78] where decreased activity of TCA cycle enzymes was reported when iron chelator (desferrioxamine) was introduced. This perhaps could be due to the removal (or unavailability in the case of IDA) of labile iron atoms from iron-sulphur clusters present in such enzymes. Systemic concentrations of leucine and isoleucine (energy sources during exercise) and methionine were also significantly reduced in our IDA piglets. These results are consistent with those reported from an 8-day neonatal piglet trial [79] in which non-iron supplemented piglets (n = 8) had lower concentrations of leucine, methionine and tyrosine compared to iron-supplemented counterparts (n = 8). Taken together, this is consistent with iron-dependent early-life metabolic shifts occurring in anaemic piglets. However, higher concentrations of urea cycle enzymes were also reported [79] which is in contrast with our results where urea cycle metabolites were significantly decreased in anaemic piglets. This difference could be a result of the 8-day trial [79] being of insufficient length for the piglets to develop severe IDA, as evidenced by reported Hb concentrations of 74.3 g/L compared to our IDA piglets (51.2 g/L). Glucose metabolism, the TCA cycle and oxidative phosphorylation are central biochemical pathways in cellular energy metabolism [80, 81],. There is evidence that the TCA cycle and iron homeostasis are linked since iron perturbations modulate the expression of Kreb’s cycle intermediates, including urea, citrulline and ornithine [82, 83]. Since decreased production of TCA cycle-associated metabolites is linked with stunting, this perhaps explains, in part, the reduced growth rate in anaemic piglets in our trial, but not the stunting observed in those piglets which received oral iron and were thus iron replete.

Host blood metabolites did not appear to be affected by the type of iron received. A plausible explanation for the observed reduction in growth rate in those piglets receiving oral iron is that luminal iron altered the composition of the gut microbiota in a manner that impacted weight gain; indeed, correlations have previously been observed between obesity and the gut microbiota [84]. Our analysis showed significant impacts of IDA and the different iron treatments on the composition of the gut microbiome, especially in core bacterial taxa. Initially we identified persistent taxa in our treatment groups which highlights the overall stability of the gut microbial communities, which occurs in pigs and humans, but not in rodents. However, although the core microbiota was largely stable in response to iron treatment, there was a lower abundance threshold for the *Bacteroides* genus in the IDA piglets compared to their iron-treated siblings. We also observed two-fold increases in abundance thresholds for *Bacteroides* in all the Oral Iron-treated piglets compared to IDA siblings which makes it a stable component of the core microbiome in these animals. This is consistent with a study conducted in 6 month old infants who received oral FeSO_4_ drops (6.6 mg Fe/day) where decreased abundance of *Bacteroides* was reported [85]. Similarly, we reported approximately two-fold decreased abundances of *Lactobacillus* in all the piglets treated with Oral Iron compared to piglets with IDA. A study in rats consuming iron-deficient diets also reported increased abundances of *Lactobacillus* along with decreased abundances of *Bacteroides* [86]. Our results appear consistent with other iron supplementation and deficiency trials which demonstrate that even if the overall core microbiota is largely stable in terms of genera, iron treatments and IDA generate consistent changes in gut microbiota composition across different species.

We identified negative correlations between *Christensenellaceae* and *Lachnospiraceae*, and total weight gain. The raised abundances of these two genera in response to oral iron supplements, combined with their associations with weight gain, raises the possibility that the reduced weight gain response to oral iron supplementation could be directly imposed by corresponding changes in the composition of the gut microbiota. Indeed, *Christensenella* abundance has been previously found to display an inverse relationship with weight gain and/or BMI in both human adults and children; increased levels of *Christensenellaceae* are strongly associated with leanness and protection against obesity as well as improved metabolic health, while lower abundance is strongly link with increased BMI and metabolic disease [87–90]. Although the underlying mechanisms are not fully understood, *Christensenella intestinihominis* MNO-863 reduced murine body weight by 10% compared to non-treated controls, and by over 15% when mice consumed high fat diets (HFDs), in a dose-dependent manner. Administration of MNO-863 also reduced hyperlipidemia, hyperglycemia, glucose and insulin resistance and non-alcoholic steatohepatitis (NASH), all obesity-related indices. Here, concurrent increases in glucagon-like peptide−1 (GLP-1) and peptide YY (PYY), both intestinal hormones, and higher concentrations of colonic propionate were also reported [91]. Similarly, daily administration of 2 × 10^9^ CFUs of a different *Christensenellaceae*, *C. minuta,* prevented hyperglycaemia and weight gain in mice consuming a HFD, despite similar feed intakes [92]. This is consistent with our study where piglets across all treatment groups consumed the same amount of feed but presented with differential weight-gains dependent on whether they received supplementary iron, and on the type of iron received. The same group showed that in a diet-induced obesity mouse model, *C. minuta* DSM33407 regulated leptin levels and hepatic lipid metabolism via inhibition of de novo lipogenesis [92]. Liu et al. also used a murine model to show that *C. minuta* drove the host to produce a previously undescribed class of secondary bile acids containing 3-O-acylation substitutions, which they termed 3-O-acyl-CAs. These inhibited the intestinal farnesoid X receptor involved in the homeostatic control of bile acids, cholesterol, fats and sugars. They subsequently demonstrated that this effect is inhibited in intestinal farnesoid X receptor knockout mice, and that 3-O-acyl-CAs were significantly reduced in humans with type 2 diabetes compared to healthy counterparts [93]. Our study focused on exploring the effects of different types of iron on the microbial populations in the gut and although *Christensenella* was strongly correlated with reductions in weight-gain in conjunction with oral iron consumption, we did not observe differences in the blood metabolites that we measured. This could be due to the tight homeostatic control of blood composition, or because such differences are associated with lipid, intestinal hormones and bile profiles (as well as colonic SCFAs), which we did not determine.

Although there are established links between increased *Christensenellaceae* abundance and weight loss, to the best of our knowledge this bacterial genus has not previously been found to be impacted by oral iron supplementation. However, iron supplementation has been linked previously with weight loss in IDA patients and this was thought to be a result of improved physical activity, reduced appetite and improved iron-related metabolic parameters as body iron levels improved [94, 95]. Here we provide compelling evidence that oral iron-induced weight loss (or reduced weight gain) could actually be a result of iron-induced modifications to the gut microbiota, specifically caused by increased abundances of *Christensenellaceae*. *Lachnospiraceae* have not been previously found to be weight-change associated but were found to be increased by oral iron treatment in postnatal pigs [96], which aligns with our findings and could also have contributed to the reduced weight gain we observed in these piglets.

It is well established that adult immune systems require large amounts of iron to operate effectively [97, 98], but less is known about the role of iron in immune development during the critical neonatal and infant phases. The studies described here thus allow novel insight into this little explored aspect of immune development within the gut. Similar to the situation in adults, reduced CD45 expression was observed in the LP of piglets with IDA compared to their IM-iron treated siblings. CD45, a receptor for tyrosine phosphatase, is highly conserved between species and is expressed on all nucleated cells of the hematopoietic system and, thus, the results are consistent with IDA piglets presenting with severe combined immune deficiency ^85^. Specifically, CD45 deficiency results in T- and B-cell dysfunction since it plays key roles in lymphocyte activation and differentiation [99]. This is especially important in young mammals which have limited immune architecture and are yet to develop a full repertoire of immune cells in response, primarily, to components of the gut microbiota. In contrast, Oral-iron only treatment resulted in significantly higher levels of CD45 in the LP at all intestinal locations investigated (compared to IM iron only controls) and increased CD45 expression has been linked with anergy and reduced survival in B-cells [100] and inhibition of T-cell receptor signalling [100]. Combined IM and oral iron treatment significantly reduced CD45 expression at all locations along the intestinal tract compared to other forms of iron treatment, although levels were not as low as for IDA piglets, which could potentially be linked to iron overload. This finding could have important implications for infants who are iron sufficient yet still receive significant quantities of oral iron via iron-fortified formula milk. It is also highly relevant to infants in LMIC who are enrolled in large scale iron supplementation programs [101, 102], even though only around 40% are iron deficient or have already developed IDA. However, as DMT1 expression was also reduced in those piglets which received both IM and oral iron, it could be that iron uptake mechanisms had been down-regulated, to reduce iron uptake from the gut lumen and thus prevent iron toxicity. This aligns with these piglets also having equivalent systemic Hb levels. Piglets which received IM iron could perhaps be considered control piglets as the IM injection of iron bypassed the gut and perhaps reflected the maternal iron endowment received during the third trimester of pregnancy. Further to this, human milk and sow milk both contain only trace amounts of iron (0.2–0.4 mg/Lt [103] and 1.4–2.6 mg/Lt [104] respectively), unlike rodent milk (7–15 mg/Lt) [105], consistent with neonatal humans and piglets naturally being exposed to only low levels of oral iron. With this in mind, it is possible that the piglets which received only oral iron expressed CD45 at much higher levels than would be normal in healthy neonates and that the piglets which received both oral and IM iron had normal levels of CD45 expression as reductions in DMT-1 expressions limited iron uptake from the lumen.

CD172 (also known as signal regulatory protein alpha; SIRPα) is a transmembrane glycoprotein primarily found on cells of myeloid origin, including dendritic cells (DCs), macrophages, granulocytes and hematopoietic stem cells. It has key roles in various immune functions including dendritic cell-mediated T cell activation, neutrophil migration and phagocytosis [106]. Our results demonstrate that IDA in piglets was associated with significant decreases in CD172^+^ populations all along the intestinal tract, compared to sibling controls which received IM iron only. Although primarily observed in blood, monocytopenia is a usual response to severe and/or prolonged IDA in human youngsters and is again indicative of a poorly functioning immune system [107]. Although oral iron supplementation has been demonstrated to reverse monocytopenia within 15 days, we show that this was not the case for piglets which received both IM and oral iron, where CD172^+^ was expressed at a similar level as in piglets which had developed IDA. Again, this is highly relevant to iron replete infants enrolled on large-scale iron supplementation programs and infants fed iron-fortified formula milk, and could play a role in the increased risk of infection in recipients of oral iron supplementation. Interestingly, we did not observe correlations between the expression of immune associated proteins and growth rate, suggesting that there is not a causal link between the two. However, we were able to link weight gain to the gut microbiota, and the immune systems develops largely in response to the gut microbiota. This could suggest that iron-induced disruption of the gut microbiota has differential effects on immune development which are highly dependent on the form of iron received.

Co-expression of CD172 and MHCII in the piglet gut mucosa primarily represents a population of the antigen presenting cells, DCs and macrophages. IDA is known to skew macrophage polarisation towards an anti-inflammatory phenotype, while iron overload favours the development of pro‐inflammatory M1 macrophages [108]. IDA is also associated with reductions in DC development and function [109], but less is understood regarding the effects of iron over-load on DC populations. However, iron overload is not anticipated in this study since 3000 mg/kg of feed is considered tolerable for piglets [110], far greater that the levels applied here. Although we cannot differentiate between macrophage and DC populations in our study, we do show that IDA in piglets led to significant reductions in the potential for antigen presentation along the intestinal tract. This was especially true in the proximal jejunum, where exposure is primarily to food-derived antigens and where tolerance to food is induced. Oral iron was consistently linked with significant increases in the potential for antigen presentation along the intestinal tract. This effect could be driven by exposure of the gut tissues to unnaturally high oral iron levels leading to perturbations in gut barrier function with a resulting increase in antigen load in the LP. Such an effect could also be mediated by modification of the gut microbiota, as recently reviewed [111]. However, providing oral iron supplementation to iron replete piglets mitigated this effect and reduced the potential for antigen presentation to similar levels to those observed in piglets with IDA. We have previously demonstrated that stromal cells are capable of presenting antigen to naïve T-cells outside of organised lymphoid tissues in the LP [112]. Here, we show that capillary endothelium expressing MHCII, presumably with the potential to present antigen, was significantly decreased in those piglets that had developed IDA and iron replete piglets which received additional oral iron, but increased in piglet that received oral iron only. Although it is difficult to determine the functional consequences of this observation without further exploration, CE has been shown to form complexes with MHCII, CD4^+^ T-cells and DCs in piglets, but not in adults pigs, and so could contribute to the development of immune tolerance during the neonatal period. Although there is some evidence that IDA diminishes antigen presenting cell function [109], to the best of our knowledge, this has not been explored in relation to different types of iron supplementation, thus we present novel findings.

This study has identified clear differences in gut immune development between piglets with IDA and those treated with iron to prevent IDA. We have previously shown that IDA in piglets leads to reduced antibody responses to vaccination [113] but we do not yet know how the observed changes in expression of immune-associated proteins in response to different forms of iron treatment might impact immune function in the infant gut, or whether the differences identified are sustained into adulthood in a manner that impacts gut immune system function in later life.

## Conclusions

This study shows that IDA causes considerable detrimental impacts on growth and immune development in neonatal-infant piglets, and also causes alterations in host metabolism and gut microbiota composition. Although iron treatment alleviated IDA, the type of iron treatment applied has distinct impacts on host development in young piglets, which are robust models for human neonates/infants. Intervention with oral iron is the most prevalent approach used to prevent the development of IDA, but these results demonstrate that such treatment causes reduced weight-gain, disruptions to the developing gut microbiota and significant changes in key immune-associated molecules in the gut mucosa, compared to IM iron-supplemented siblings. Importantly, we provide compelling evidence, for the first time, which links increased luminal iron to significant increases in abundances of *Lachnospiraceae* and *Christensenella* taxa*,* which we have correlated with inadequate weight-gain in piglets. If these findings are reflected in human infants, they will have important implications for iron intervention strategies aimed at combating IDA, especially in LMICs, where infant nutrition may already be insufficient to ensure appropriate weight-gain. The findings reported here also have important implications for formula-milk fed infants, especially for those that are iron replete since formula milk is routinely fortified with iron, even for those infants where the requirement for such fortification is debatable and may impose negative health consequences, as highlighted here.

## Supplementary Information

Below is the link to the electronic supplementary material.

Supplementary file 1

## Data Availability

The data that support the findings of this study are available from the corresponding author, Marie C Lewis, upon reasonable request. The 16S rRNA gene sequencing raw data with metadata have been deposited in the NCBI database (accession number: PRJNA1428132).
