# Supplementary material for: Oral iron-induced stunting is associated with increased Christensenella abundance in a piglet model for human infants
Source: Eur J Nutr. 2026 Aug 27;65(6):243. doi: 10.1007/s00394-026-04082-9 (PMC13521992; doi:10.1007/s00394-026-04082-9)
Supplement: Supplementary file 1 [file 394_2026_4082_MOESM1_ESM.docx]

**Table S1.** Pairwise beta diversity comparison of all four treatment groups

|  | Groups  (Pairwise combination) | **Bray Curtis** | | **UniFrac** | | **Weighted UniFrac** | |
| --- | --- | --- | --- | --- | --- | --- | --- |
|  |  | R^2^ | P | R^2^ | p | R^2^ | p |
| 1 | IDA vs IM Iron | 0.06686 | NS | 0.06616 | NS | 0.03902 | NS |
| 2 | IDA vs Oral Iron | 0.15889 | 0.003 ** | 0.13058 | NS | 0.0888 | NS |
| 3 | IDA vs IM Iron + Oral Iron | 0.12459 | 0.033 * | 0.10542 | NS | 0.09009 | NS |
| 4 | IM Iron vs Oral Iron | 0.10131 | 0.001 *** | 0.07792 | NS | 0.04907 | NS |
| 5 | IM Iron vs IM Iron + Oral Iron | 0.13512 | 0.006 ** | 0.11233 | NS | 0.0904 | NS |
| 6 | Oral Iron vs IM Iron+ Oral Iron | 0.11244 | NS | 0.11639 | NS | 0.09674 | NS |

**Figure S1.** The bacterial genera shared between all treatment groups extracted using core microbiome analysis.
